# A sequentially triggered DNA nanocapsule for targeted drug delivery based on pH-responsive i-motif and tumor cell-specific aptamer

**DOI:** 10.3389/fbioe.2022.965337

**Published:** 2022-08-25

**Authors:** Baoyin Yuan, Yanan Xi, Cuihua Qi, Mingzhu Zhao, Xiaoyan Zhu, Jinlu Tang

**Affiliations:** School of Basic Medical Sciences, Zhengzhou University, Zhengzhou, Henan, China

**Keywords:** DNA nanocapsule, pH-responsive i-motif, aptamer recognition, targeted drug delivery, sequentially triggered release

## Abstract

Targeted drug delivery with minor off-target effects is urgently needed for precise cancer treatments. Here, a sequentially triggered strategy based on double targeting elements is designed to meet this purpose. By using an acidic pH-responsive i-motif DNA and a tumor cell-specific aptamer as targeting elements, a smart dual-targeted DNA nanocapsule (ZBI5-DOX) was constructed. ZBI5-DOX can be firstly triggered by acidic pH, and then bind to target cells via aptamer recognition and thus targeted release of the carried DOX chemotherapeutics. With this smart DNA nanocapsule, the carried DOX could be precisely delivered to target SMMC-7721 tumor cells in acidic conditions. After drug treatments, selective cytotoxicity of the DNA nanocapsule was successfully achieved. Meanwhile, the DNA nanocapsule had a specific inhibition effect on target cell migration and invasion. Therefore, this sequentially triggered strategy may provide deep insight into the next generation of targeted drug delivery.

## Introduction

Targeted drug delivery, which aims to precisely deliver drugs to cancer cells, has reformed cancer therapies with improved therapeutic efficacy and alleviative side effects (Dugger et al., 2018; Malone et al., 2020). Especially, with the emergence of nanomedicine, targeted drug delivery extremely facilitates the development of cancer therapy (Kim et al., 2019; Manzari et al., 2021). Ligand-targeted drug delivery is the most common strategy among the various targeted drug delivery methods. Ligands can selectively bind to the overexpressed receptors on tumor cells and promote drug internalization, resulting in accumulated drugs inside the tumor cells and reduced harm to healthy cells (van der Meel et al., 2013; Srinivasarao et al., 2015). For a long time, ligands have mainly relied on antibodies, peptides and small organic molecules, which may have some limitations, such as difficulties in modification and design, high costs and biotoxicity (Yao et al., 2016; Srinivasarao and Low, 2017).

Aptamers are short single-stranded DNA or RNA sequences evolved through SELEX (systematic evolution of ligands by exponential enrichment), which can specially bind to targets with high affinity (Ellington and Szostak, 1990; Tuerk and Gold, 1990). As emerging recognition ligands, aptamers displayed unique advantages over conventional ligands, such as non-toxicity, easy chemical synthesis, controllable modification, and flexible design with DNA nanotechnology (Tan et al., 2013; Ma et al., 2015; Dunn et al., 2017; Yuan et al., 2017). It is elegant to apply tumor cell-specific aptamers as targeting ligands for targeted drug delivery. In recent years, aptamer-based targeted drug delivery has been widely explored (Yoon and Rossi, 2017; Zhu and Chen, 2018). By using aptamers as targeting ligands, different designs or formulations of aptamer-based drug delivery systems have been successfully made for improving targeted cancer therapies, such as aptamer-drug conjugates, aptamer-therapeutic oligonucleotide conjugates, and aptamer-decorated nanomaterial systems (Pastor et al., 2010; Liang et al., 2015; Li et al., 2017; Zhou and Rossi, 2017; Huang et al., 2021).

Although ligand-targeted drug delivery is efficient, pH-responsive targeting is a promising method for targeted drug delivery. According to the reports, tumors presented an acidic extracellular pH, which has been used as an attractive target for targeted drug delivery (Weis and Cheresh, 2011; Belli et al., 2018). The past decades have witnessed various strategies for acidic pH-responsive targeted drug delivery including small organic molecules, biomacromolecules, liposomes, micelles and polymeric nanoparticles (Danhier et al., 2010; Kanamala et al., 2016). I-motif DNA are cytosine-rich sequences that can form a tetraplex via a stack of intercalating hemiprotonated cytosine-neutral cytosine base pairs at acidic pH (Zeraati et al., 2018). The responsive pH range of i-motif DNA could be accurately regulated by changing the C-tract length and i-loop sequences of the i-motif DNA (Lei et al., 2018; Debnath et al., 2019). Notably, due to the nature of nucleic acids, i-motif DNA have the inherent advantages of good biocompatibility, easy synthesis, functional modifications, and flexible integration with aptamers, DNA blocks and nanostructures (Dong et al., 2014; Ma et al., 2019; Mergny and Sen, 2019). These merits enable i-motif DNA as an ideal targeting element for construction of pH-responsive drug delivery systems.

**SCHEME 1:**
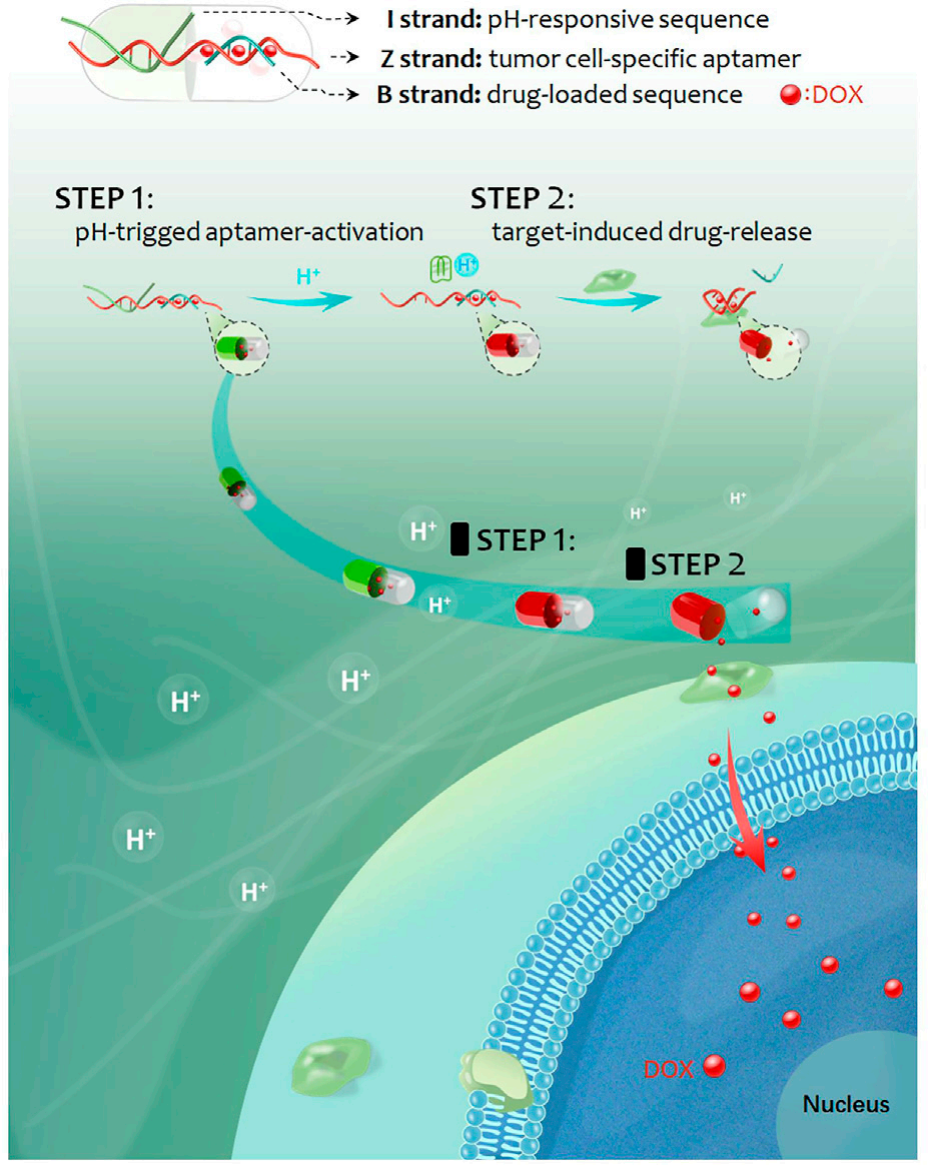
Schematic representation of the sequentially triggered DNA nanocapsule for targeted drug delivery.

As reported, most of the strategies of targeted drug delivery rely on single targeting element, which easily lead to off-target drug delivery (Rosenblum et al., 2018; Gu et al., 2021). However, sequentially triggered strategy based on double targeting elements is probably able to improve this issue. Herein, by using an acidic pH-responsive i-motif DNA and a tumor cell-specific aptamer as molecular targeting tools, a smart sequentially triggered DNA nanocapsule was designed and constructed for targeted drug delivery. As shown in Scheme 1, the DNA nanocapsule was consisted of three components, acidic pH-responsive i-motif DNA sequences (I strand), tumor cell-specific aptamer (Z strand) and drug-loaded carrier sequences (B strand). I strand was hybridized with 3’ end region of Z strand for blocking recognition ability of Z strand. B strand was hybridized with middle region of Z strand for carrying DOX chemotherapeutics. When the target tumor cells were treated with the DNA nanocapsule in acidic conditions, acidic pH would trigger the dissociation of I strand from the DNA nanocapsule, and then the recognition ability of Z strand to target cells would recover and the drug loading region would be destroyed, and thus sequential targeting-induced release of carried DOX and targeted killing of tumor cells by DOX.

## Materials and methods

### Chemicals and materials

All DNA were synthesized and purified by Sangon Biotechnology Company, Ltd. (Shanghai). Sequences of all the DNA were listed in Supplementary Table S1. Dulbecco’s phosphate buffered saline (D-PBS) was purchased from Corning. 0.1 M PB buffer was prepared using NaH_2_PO_4_ and Na_2_HPO_4_. Binding buffer was D-PBS containing 5 mM MgCl_2_, 4.5 g/L glucose, 1 mg/ml BSA and 0.1 mg/ml yeast tRNA. Methylthiazolyldiphenyl-tetrazolium bromide (MTT), yeast tRNA, bovine serum albumin (BSA) and propidium iodide (PI) were purchased from Sigma-Aldrich. Calcein-AM, SYBR Gold and Hoechest 33,342 were purchased from Thermo Fisher Scientific. Doxorubicin (Dox) was purchased from Dingguo Biotechnology Company, Ltd. (Beijing). Caspase 3 detection kit was purchased from Beyotime Biotechnology Company, Ltd. (Shanghai). Annexin-V FITC apoptosis detection kit was purchased from Sangon Biotechnology Company, Ltd. (Shanghai). Other chemicals were of analytical grade and used without further purification.

### Cells

Hepatocellular carcinoma SMMC-7721 and HepG-2 cells, cervical carcinoma HeLa cells, esophageal carcinoma KYSE30 cells and hepatocyte HL-7702 cells used in the experiment were purchased from Cell Bank of the Committee on Type Culture Collection of the Chinese Academy of Sciences (Shanghai, China). Gastric carcinoma BGC-823 cells, pancreatic carcinoma KP-4 and PCNA-1 cells were offered by our laboratory. All the cells were human-derived. The Cells were cultured in RPMI 1640 with 10% fetal bovine serum (FBS) at 37°C in a humidified incubator containing 5 wt%/vol CO_2_. Both subculture and pretreatment of cells were completed in the clean bench.

### Preparation and characterization of DNA nanocapsule

Different oligonucleotides were dissolved in D-PBS and heated at 95°C for 5 min, then cooled at 4°C for 10 min. Subsequently, the oligonucleotides in different proportions were mixed in D-PBS at room temperature for at least 40 min for DNA hybridization. For construction of DNA nanocapsule, DOX was incubated with the above DNA complex (ZB) for drug loading. Then, the ZB-DOX was used for further assembly of ZBI5, which loaded DOX and called ZBI5-DOX. The formation of DNA nanocapsule were characterized by 10% native PAGE, the UV-visible spectrophotometer (Hitachi, Japan) and the F-7000 fluorescence spectrophotometer (Hitachi, Japan).

### Flow cytometry assays

Generally, 100,000 cells were resuspended in 200 μl of binding buffer, then different DNA sequences or assembles were added and incubated at 25°C for 25 min, and immediately analyzed by flow cytometer (FACSCanto II, BD Biosciences) by counting 10,000 events. For evaluating performance of DNA nanocapsule in complex condition, 10% fetal bovine serum was added into binding buffer for flow cytometry assays. For investigating the specific recognition of DNA nanocapsule in mixed cells, target SMMC-7721 cells were incubated with 4 μM Calcein-AM at 37°C for 20 min, then washing with binding buffer for three times. Subsequently, the same amount of target SMMC-7721 cells and control HepG-2 cells were mixed in 200 μl binding buffer, after incubated with 100 nM ZB5 probes at 25°C for 25 min, the cells were subjected to flow cytometry assays.

For assessing targeted uptake of DNA nanocapsule, the cells were treated with 3 μM of DOX or ZBI5-DOX in RPMI 1640 medium without FBS at 37°C for 4 h, then the cells were washed with PBS for three times and digested by trypsin, after washing and centrifugation, the cells were resuspended in 500 μl PBS and subjected to DOX fluorescence analysis by flow cytometry. For apoptosis analysis, after treated with DOX or ZBI5-DOX, the cells were incubated with 5 μl Annexin-FITC and 10 μl PI at room temperature for 15 min, then followed by flow cytometry analysis. For caspase-3 detection, after drug treatments, the cells were digested and washed, and incubated with 5 μM of detecting substrate of caspase-3 for 30 min, then analyzed by flow cytometry.

### Confocal imaging

SMMC-7721 and HepG-2 cells were cultured overnight in dishes, removed the culture medium and washed with PBS twice. The cells were then incubated with DNA nanocapsule (300 nM) in binding buffer at 25°C for 30 min. After incubation, the cells were washed with PBS for three times and imaged by a laser scanning confocal microscope (LSCM, Olympus, Japan). For assessing targeted uptake of DNA nanocapsule, the cells were treated with 3 μM of DOX or ZBI5-DOX in 1640 medium without fetal bovine serum at 37°C for 4 h, after washing with PBS for three times, the cells were stained with Hoechst33342 at 37°C for 15 min, then imaged by the LSCM after washed with PBS for three times. For analyzing cell viability, the cells were subjected to the same drug treatments and cultured in 1640 medium (containing 10% FBS) for 48 h, after washing, the cells were incubated with 0.1 μg/ml Calcein-AM and 1 μg/ml PI in PBS at 37°C for 30 min, then washing for three times and followed by LSCM imaging. Fluorescence channel: for Hoechst33342, EX 405 nm, EM 435–460 nm; for Alexa 488, EX 488 nm, EM 525 nm long-pass; for Calcein-AM, EX 488 nm, EM 505–525 nm; For Cy5, EX 635 nm, EM 660 nm long-pass.

### Methylthiazolyldiphenyl-tetrazolium bromide assay

SMMC-7721 and HepG-2 cells (8,000 cells/well) were seeded into 96-plates and cultured overnight, the cells were incubated with different concentration of DOX (0, 0.5, 1, 2 and 3 μM) for 4 h, removed the old medium, added new medium and cultured for 48 h. MTT (5 mg/ml, 20 μL/well) was added to each well, and the plate was incubated at 37°C for 4 h, removed the supernatant and added 200 μl DMSO into each well, shaked for 10 min and detected the OD value at 490 nm by a microplate reader.

### Wound-healing assay

Cells (3 × 10^5^/well) were plated in 12-well plates. After culturing for 12 h, cells were wounded with a sterile 10 μl pipette tip and washed with PBS for removing dissociated cells. Then the cells were cultured in serum-free medium. Cell images were taken at the same position of the wound every 24, 48 and 72 h by a phase-contrast microscopy. The areas of the wounds were measured using ImageJ.

### Transwell assay

Cells (1 × 10^5^) in 200 μl serum-free medium were seeded into the upper chamber with Materigel (BD, United States), and 800 μl medium with 20% FBS were added into the lower chamber. After culturing for 24 h, the cells were fixed with methanol for 30 min and stained with 0.2% crystal violet for 20 min at room temperature. After washing, the noninvaded cells on the upper side were cleared, the invaded cells on the bottom side were remained. Cell images were obtained by a microscope, and cell number was calculated through three random microscopic fields and averaged.

### Statistical analysis

Statistical analysis was performed by one-way analysis of variance or non-parametric tests using SPSS version 22.0 (SPSS). The quantitative results are expressed as the mean ± standard deviation, and *p* < 0.05 were considered statistically significant.

## Results and discussion

### Investigation of pH responsivity of ZI complex

**FIGURE 1:**
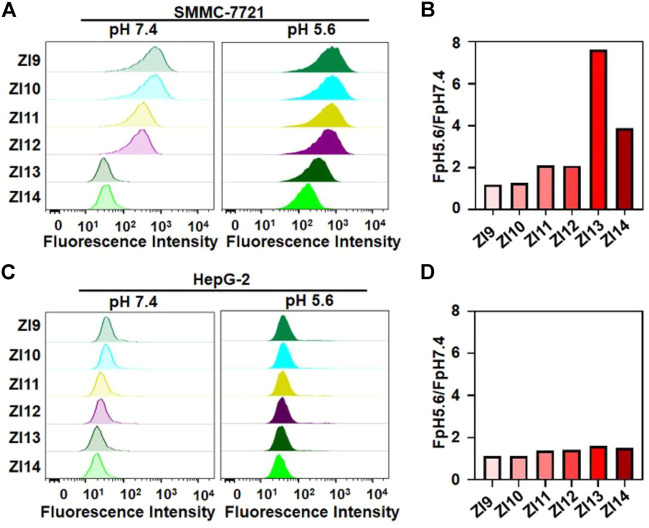
Investigation of binding performance of ZI9-14 in binding buffer. Flow cytometric analysis of SMMC-7721 **(A)** and HepG-2 **(C)** at pH 5.6 and 7.4 after treatment with 50 nM probes for 25 min at 25°C, respectively. **(B)** and **(D)** Corresponding histogram of the fluorescence ratios of pH 5.6 to pH 7.4 for probes in **(A)** and **(C)**, respectively.

For designing the DNA nanocapsule, the combination between aptamer sequences and i-motif DNA (ZI complex) was firstly investigated. As proof of concept, a Z11 DNA aptamer that specifically bind to SMMC-7721 cells was applied (Tang et al., 2020; Yuan et al., 2020). Since the stability of double-strand region between I strand and Z strand determined pH-responsive conformation of I strand, the base-pair number of double-strand region was optimized. Six I strand labeled with BHQ1 quenching group (I9-I14, the number denoted complementary base pairs, Supplementary Table S1) were designed to hybridize with Z strand labeled with Alexa 488 fluorescent group (pH-insensitive, Supplementary Figure S1). The formed double-strand DNA complex were respectively termed as ZI9-ZI14, which showed low fluorescence because of close distance between BHQ1 and Alexa 488. When meet acidic conditions, I strand dissociated from the double-strand DNA and Alexa 488 was away from BHQ1, and Alexa 488 fluorescence recovered. As shown in Supplementary Figure S2, I strand displayed decreasing responsive pH range and median with the increasing base-pair number between I strand and Z strand. To obtain the optimum ZI, ZI9-ZI14 were used to incubated with target SMMC-7721 and control HepG-2 cells in an acidic condition, the results of flow cytometry assays showed that ZI13 had the highest signal intensity for target cell recognition compared to control cells (Figure 1). Fluorescence spectrum also showed that ZI13 respond to acidic pH effectively, but cZI13, a negative control probe of ZI13, showed negligible fluorescence response (Supplementary Figure S3). Therefore, ZI13 was selected for subsequent experiments.

### Acidic pH-triggered binding of ZI13

**FIGURE 2:**
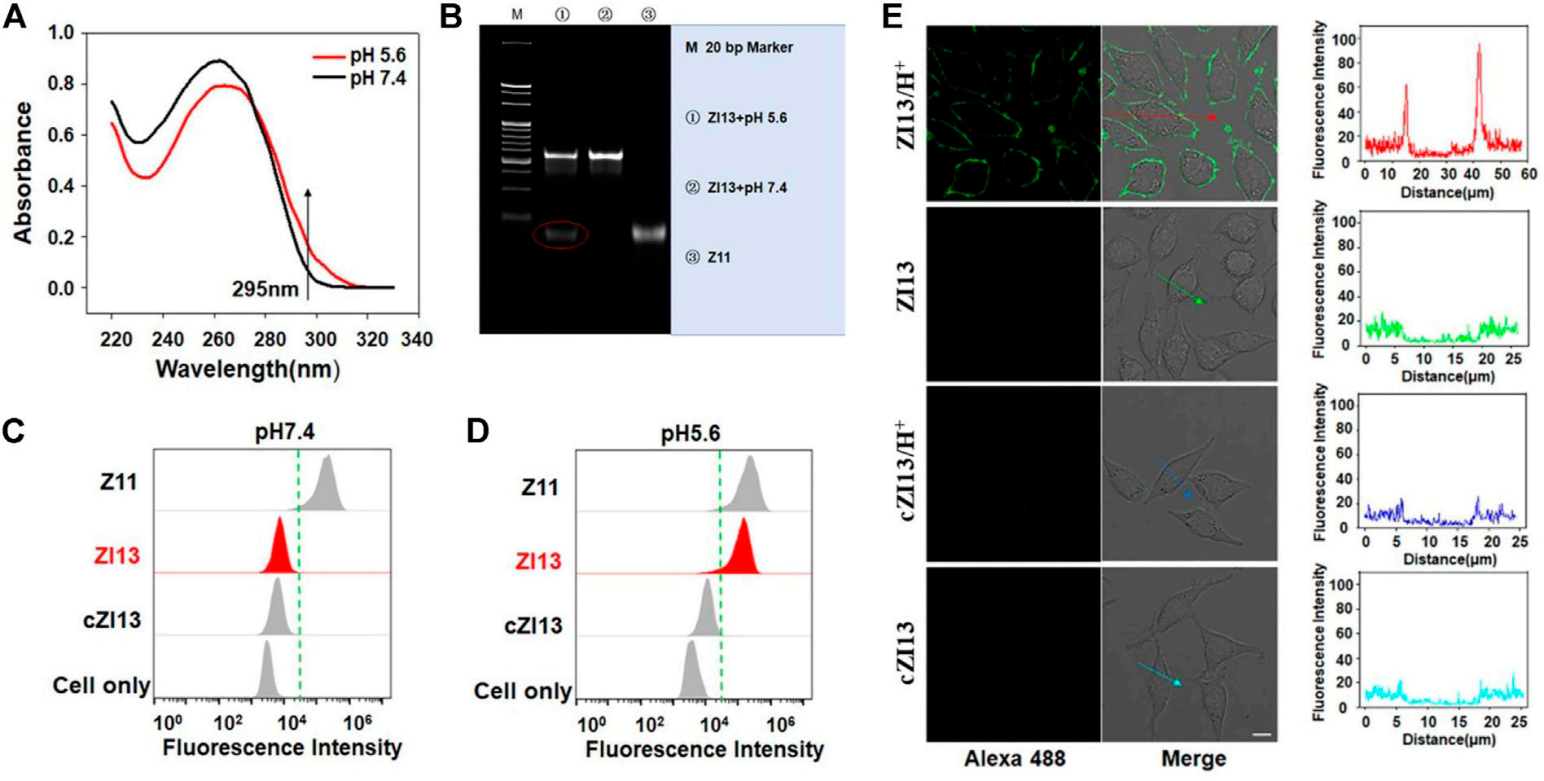
Characterization of ZI13. **(A)** UV spectrum of ZI13. The arrow indicates absorbance at 295 nm. **(B)** PAGE images of ZI13 at pH 5.6 (lane 1), ZI13 at pH 7.4 (lane 2) and Z11 (lane 3). Flow cytometry analysis of binding ability of ZI13 to target SMMC-7721 cells at pH 7.4 **(C)** and pH 5.6 **(D)**. Z11 was used as positive controls and cZI13 was used as negative controls. **(E)** LSCM images of target SMMC-7721 cells incubated with ZI13 and cZI13 at pH 5.6 and pH 7.4. The fluorescence intensity indicated by the arrows was listed right.

To verify the formation of i-motif structure of ZI13 under acidic conditions, the UV spectrum was recorded. As seen in Figure 2A, the characteristic absorbance of i-motif of ZI13 at 295 nm increased significantly when the pH decreased from 7.4 to 5.6, similar trend was observed in I13 positive control (Supplementary Figure S4A), while cI13 (cytosine-rich region of I13 was replaced by thymine, Supplementary Figure S4B) and cZI13 (cI13 hybridizing with Z strand, Supplementary Figure S4C) negative controls showed no changes, these results suggested successful formation of i-motif at acidic conditions. PAGE image further confirmed the dissociation of I13 from ZI13 when i-motif was formed at acidic conditions (Figure 2B), while cI13 could not dissociated from cZI13 (Supplementary Figure S5). To obtain higher signal-to-background ratio (SBR) of ZI13 to target cells, the ratio of I13 to Z11 and incubation time were optimized, respectively. The results demonstrated that the SBR was highest when the ratio of I13 to Z11 was 2.5 and incubation time was 25 min (Supplementary Figure S6), which were used for subsequent experiments. The acidic pH-responsive binding ability of ZI13 to target SMMC-7721 cells was tested by flow cytometry, as shown in Figure 2C, ZI13 did not bind target cells at pH 7.4, but significant binding at pH 5.6 (Figure 2D), indicating great binding ability of ZI13 to target cells at acidic conditions. Confocal imaging was used to further evaluate the acidic pH-responsive binding ability of ZI13, as displayed in Figure 2E, significant fluorescence signal was only observed on target cells incubated with ZI13 in acidic conditions, but not on target cells in pH 7.4 and control HepG-2 cells in acidic conditions (Supplementary Figure S7), further proved the acidic pH-responsive binding ability of ZI13 to target cells. Furthermore, to assess the binding specificity of ZI13, five control cell lines were analyzed by flow cytometry. The results showed no significant binding of ZI13 to these five cell lines (Supplementary Figure S8), indicating excellent binding specificity of ZI13.

### Aptamer-triggered conformation changes

**FIGURE 3:**
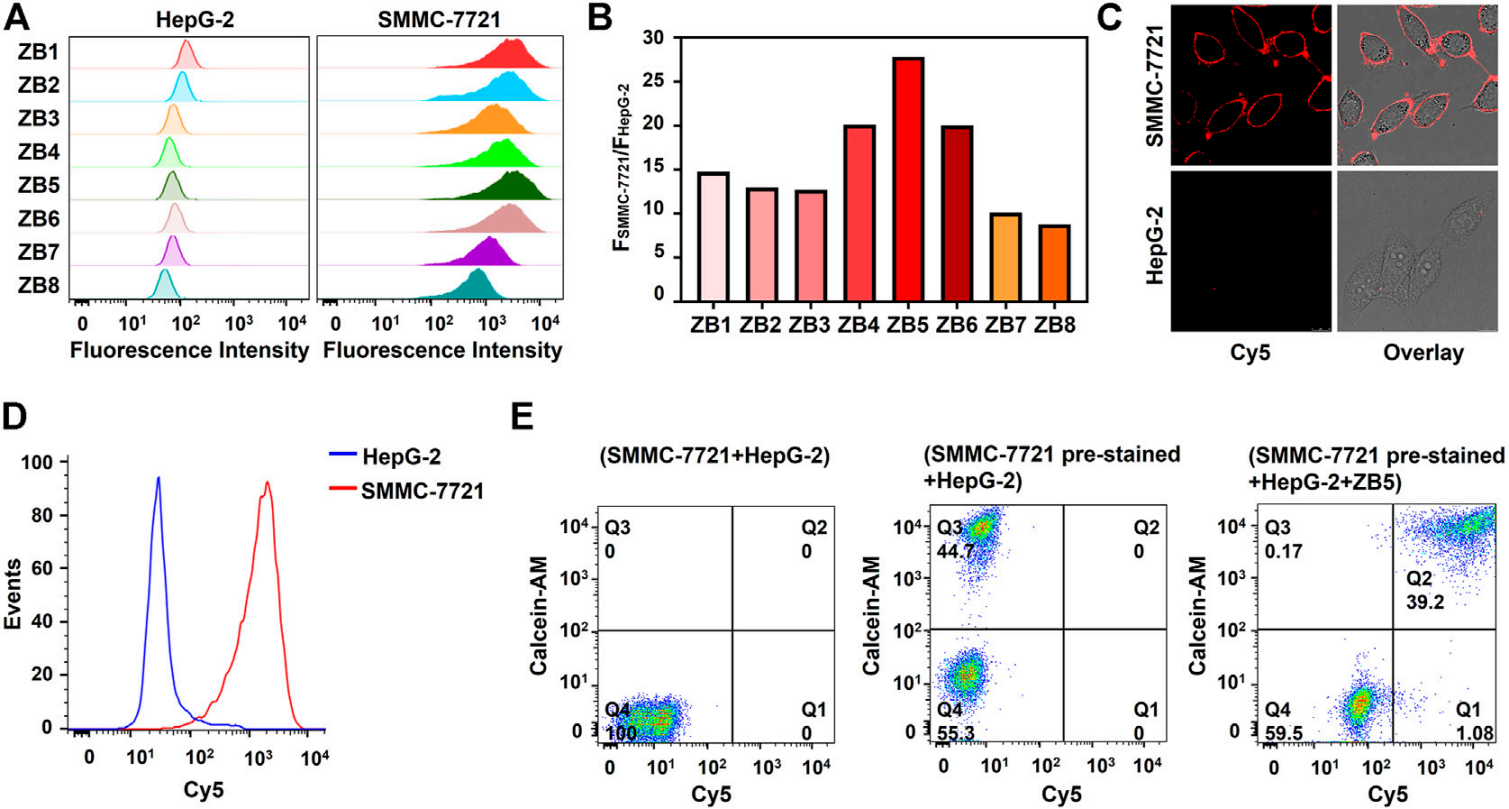
Optimization and characterization of ZB complex. **(A)** Flow cytometry assays of SMMC-7721 and HepG-2 cells incubated with ZB1-8. **(B)** Corresponding SBR of Figure **(A)**. SBR represents the ratio of fluorescence intensity of SMMC-7721 cells to HepG-2 cells. **(C)** Confocal imaging of SMMC-7721 and HepG-2 cells incubated with ZB5. **(D)** Flow cytometry assays of SMMC-7721 and HepG-2 cells incubated with ZB5 in complex conditions. **(E)** Flow cytometry assays of recognition ability of ZB5 for mixed cell samples. Calcein-AM was used as pre-stained dyes.

For designing the smart DNA nanocapsule, B strand was hybridized with Z strand for carrying DOX. The formed double-strand DNA complex was termed as ZB complex. Because the stability of double-strand region of ZB determined the recognition of Z strand to target cells and the releasing of DOX, the number of base-pairs between Z strand and B strand was investigated. BHQ2-labeled B1-8 with 8–15 bases complementary to Cy5-labeled Z strand were respectively designed to construct ZB1-8. ZB displayed negligible fluorescence signal when target cells were absent, while showed strong fluorescence signal when target cells were present due to recognition-induced dissociation of BHQ2-labeled B strand from ZB. As shown in Figure 3A, with increasing number of base-pairs, target SMMC-7721 cells showed decreased fluorescence signal after incubated with ZB1-8, the results of corresponding SBR displayed that ZB5 with 12 base-pairs to Z11 strand had the highest SBR (Figure 3B), which was selected for constructing the smart DNA nanocapsule. Confocal imaging was used to visualize the specific recognition of ZB5 to target cells. As displayed in Figure 3C, strong fluorescence signal was observed on target SMMC-7721 cells, but not on control HepG-2 cells, verified the specific binding of ZB5 to target cells.

To further improve SBR, the ratio of B5 to Z11 and incubation time of ZB5 to target cells were optimized. The results showed that SBR were highest when the ratio of B5 to Z11 was 1.5 and incubation time was 25 min (Supplementary Figure S9), which was used for subsequent experiments. The binding specificity of ZB5 was also evaluated by flow cytometry, the results displayed that ZB5 only bind to target SMMC-7721 cells rather than four control cells (Supplementary Figure S10), suggested great binding specificity of ZB5 to target cells. Moreover, the binding ability of ZB5 in complex conditions was further investigated. 10% FBS was added into binding buffer for simulating complex conditions. Flow cytometry assays showed that ZB5 could bind to target cells strongly (Figure 3D), indicating excellent binding ability of ZB5 in complex conditions. Furthermore, the recognition ability of ZB5 for mixed cells was assessed. The mixed cells were consisted of target SMMC-7721 cells pre-stained with Calcein-AM and control HepG-2 cells. After incubated with ZB5, target cells were observed in upright positive area, while control cells were observed in bottom-left negative area (Figure 3E), indicated excellent recognition ability of ZB5 to target cells in mixed cells.

### Construction and characterization of the DNA nanocapsule

**FIGURE 4:**
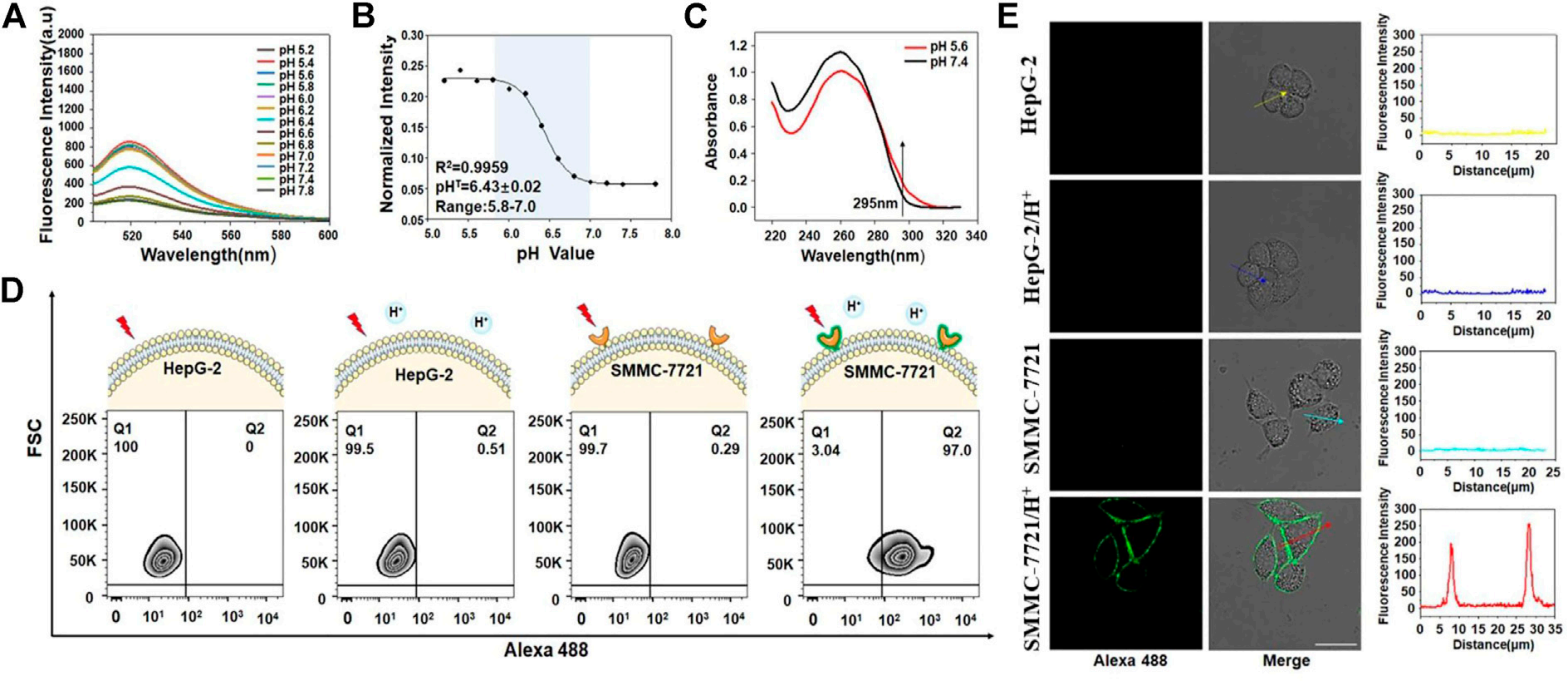
Construction and characterization of ZBI5. **(A)** Fluorescence spectrum of ZBI5 (100 nM) in pH 5.2–7.8. **(B)** Fluorescence profile of ZBI5 (100 nM) in pH 5.2–7.8. The normalized intensity represents the ratio of fluorescence intensity of ZBI5 to Alexa 488-labeled Z11. pH^T^ indicates responsive pH median. **(C)** UV absorbance spectrum of ZBI5 at pH 5.6 and 7.4. The arrow indicates absorbance at 295 nm. **(D)** Flow cytometry assays of recognition of ZBI5 to target SMMC-7721 and control HepG-2 cells in acidic (pH 5.6) and non-acidic (pH 7.4) conditions. **(E)** LSCM images of target SMMC-7721 cells and control HepG-2 cells incubated with ZBI5 in pH 5.6 and 7.4. The fluorescence intensity indicated by the arrows was listed right.

Because ZI13 and ZB5 had been successfully constructed, the building blocks of ZI13 and ZB5 (Z11, I13 and B5) were used to construct DNA nanocapsule. The constructed DNA nanocapsule was named as ZBI5. To test the responsive behavior of ZBI5 in acidic conditions, the fluorescence intensity of ZBI5 in pH 5.2–7.8 was recorded. From the results, ZBI5 showed negligible fluorescence changes in pH 7.0–7.8, while significant fluorescence changes in acidic conditions compared to cZBI5 control (Figures 4A and Supplementary Figure S11), the fluorescence profile of ZBI5 also displayed sensitive responses in acidic conditions (Figure 4B). To obtain a higher and more stable signal, plateau pH 5.6 was selected as an acidic condition for characterization of ZBI5. UV spectrum showed that the absorbance of ZBI5 at 295 nm increased significantly when the pH decreased from 7.4 to 5.6 (Figure 4C), while cZBI5 control had no similar trend (Supplementary Figure S12), these results indicated the formation of i-motif structure of ZBI5 in acidic conditions.

To improve ZBI5 targeting to SMMC-7721 cells, the incubation concentration and time were optimized. The results demonstrated that SBR were highest when the incubation concentration was 100 nM and incubation time was 25 min (Supplementary Figure S13), which were used for subsequent experiments. The specific recognition of ZBI5 to target cells was further evaluated by flow cytometry. As seen in Figure 4D, 97% of target SMMC-7721 cells were detected in positive area (Q2) after indicated with ZBI5 in acidic conditions, while target cells incubated with ZBI5 in pH 7.4, control HepG-2 cells incubated with ZBI5 were not substantially detected in Q2, and control cZBI5 also showed no significant recognition to target and control cells (Supplementary Figure S14), these results suggested distinctive recognition ability of ZBI5 in acidic conditions. Confocal imaging was used to confirm the specific recognition of ZBI5. As shown in Figure 4E, bright fluorescence was only observed on target SMMC-7721 cells incubated with ZBI5 in acidic conditions, while target cells incubated with ZBI5 in pH 7.4, target cells incubated with cZBI5 and control HepG-2 cells displayed no fluorescence (Supplementary Figure S15), further verified the specific recognition of ZBI5.

### Drug loading

**FIGURE 5:**
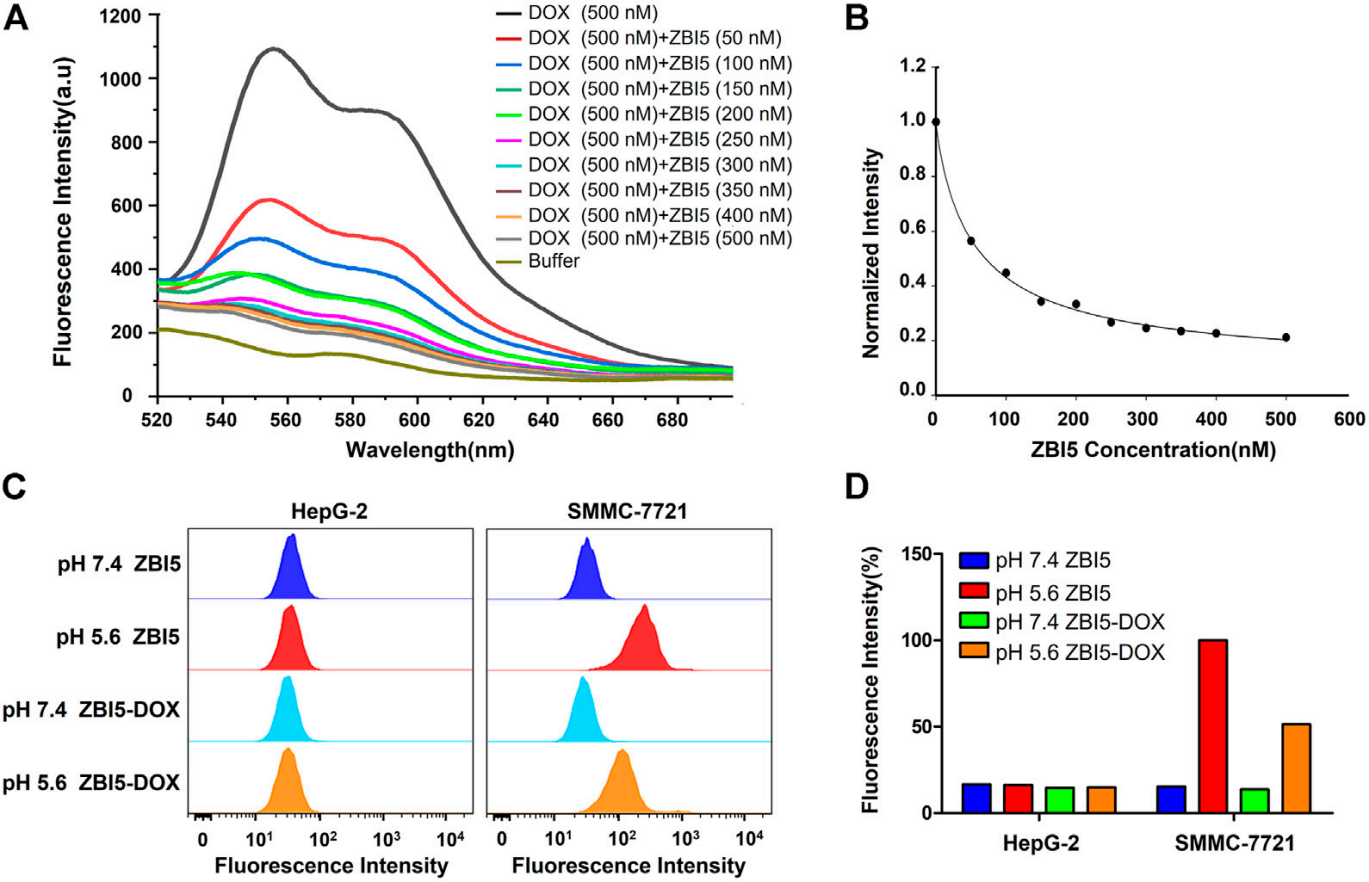
Fluorescence spectrum **(A)** and the normalized fluorescence intensity **(B)** of Dox solutions added with ZBI5 at different ratio of ZBI5 to Dox. The ratio of ZBI5 to Dox was 0, 0.1, 0.2, 0.3, 0.4, 0.5, 0.6, 0.7, 0.8 and 1.0. Flow cytometry assays **(C)** and the corresponding normalized fluorescence intensity **(D)** of target SMMC-7721 and control HepG-2 cells incubated with ZBI5 and ZBI5-DOX in pH 5.6 and 7.4, respectively.

DOX was loaded into ZB5 (ZB5-DOX) through intercalating with GC base at room temperature, the ZB5-DOX was then hybridized with functional DNA building components named I13 for further construction of ZBI5-DOX. Since fluorescence of the loaded DOX was quenched, decreased fluorescence spectrum of ZBI5-DOX was recorded to verify DOX loading. As shown in Figures 5A,B, the fluorescence signal of DOX gradually decreased with the increased concentration of ZBI5, indicating successful drug loading of the DNA nanocapsule. And a saturated DOX loading was obtained at a molar ratio of ∼0.6, which was applied to the subsequent targeted treatments. To evaluate whether the loaded Dox would affect the recognition of ZBI5 to target cells, flow cytometry was used to test the binding ability of ZBI5-DOX. As displayed in Figures 5C,D, ZBI5-DOX showed comparable fluorescence signal in acidic conditions compared to ZBI5, and could not recognize target cells in pH 7.4. Meanwhile, cZBI5-DOX control showed no specific recognition to target cells in acidic conditions (Supplementary Figure S16). These results implied that DOX loading did not significantly affect the recognition ability of ZBI5 to target cells.

### Targeted drug delivery

**FIGURE 6:**
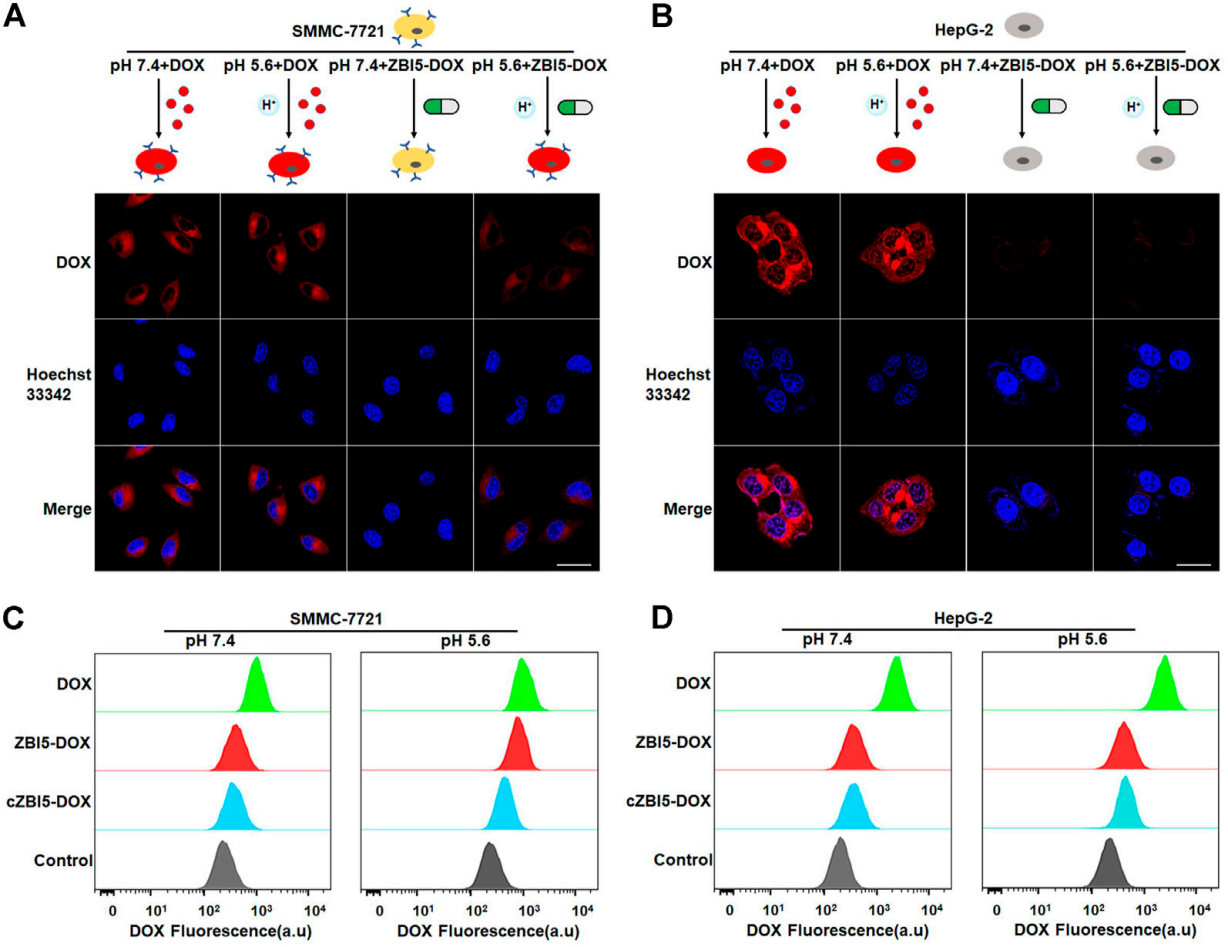
Targeted drug delivery of the smart DNA nanocapsule. **(A)** Confocal images of target SMMC-7721 cells incubated with free DOX and ZBI5-DOX in pH 5.6 and 7.4. **(B)** Confocal images of control HepG-2 cells incubated with free DOX and ZBI5-DOX in pH 5.6 and 7.4. **(C)** Flow cytometry assays of target SMMC-7721 cells incubated with free DOX, cZBI5-DOX and ZBI5-DOX in pH 5.6 and 7.4. **(D)** Flow cytometry assays of control HepG-2 cells incubated with free DOX, cZBI5-DOX and ZBI5-DOX in pH 5.6 and 7.4.

The targeted drug delivery of ZBI5-DOX to target SMMC-7721 cells was verified by using confocal imaging. As demonstrated in Figures 6A,B, strong fluorescence was observed in target SMMC-7721 cells incubated with ZBI5-DOX in acidic conditions, but not in target cells in pH 7.4 and control HepG-2 cells in pH 5.6 and 7.4. In contrast, free DOX nonselectively permeated into both target and control cells in pH 5.6 and 7.4. These results suggested that only target cells in acidic conditions could trigger the releasing of DOX in ZBI5-DOX, which enabled targeted drug delivery of ZBI5-DOX. In addition, flow cytometry was used to further verify the targeted drug delivery of ZBI5-DOX to target cells. As shown in Figure 6C and Supplementary Figure S17A, the fluorescence intensity of target SMMC-7721 cells incubated with ZBI5-DOX in acidic conditions was significantly higher than that of target cells in pH 7.4 and target cells incubated with cZBI5-DOX, and free DOX displayed a nonselective high fluorescence intensity in target cells in pH 5.6 and 7.4. Moreover, control HepG-2 cells showed a low fluorescence intensity to both ZBI5-DOX and cZBI5-DOX in pH 5.6 and 7.4 (Figure 6D and Supplementary Figure S17B). Meanwhile, the control pH-responsive delivery group (ZI-DOX) and target-activated delivery group (ZB-DOX) displayed nonselective fluorescence intensity in non-target samples (Supplementary Figures S18,S19). From these results, we confirmed the targeted drug delivery of ZBI5-DOX to target cells.

### *In vitro* selective cytotoxicity

**FIGURE 7:**
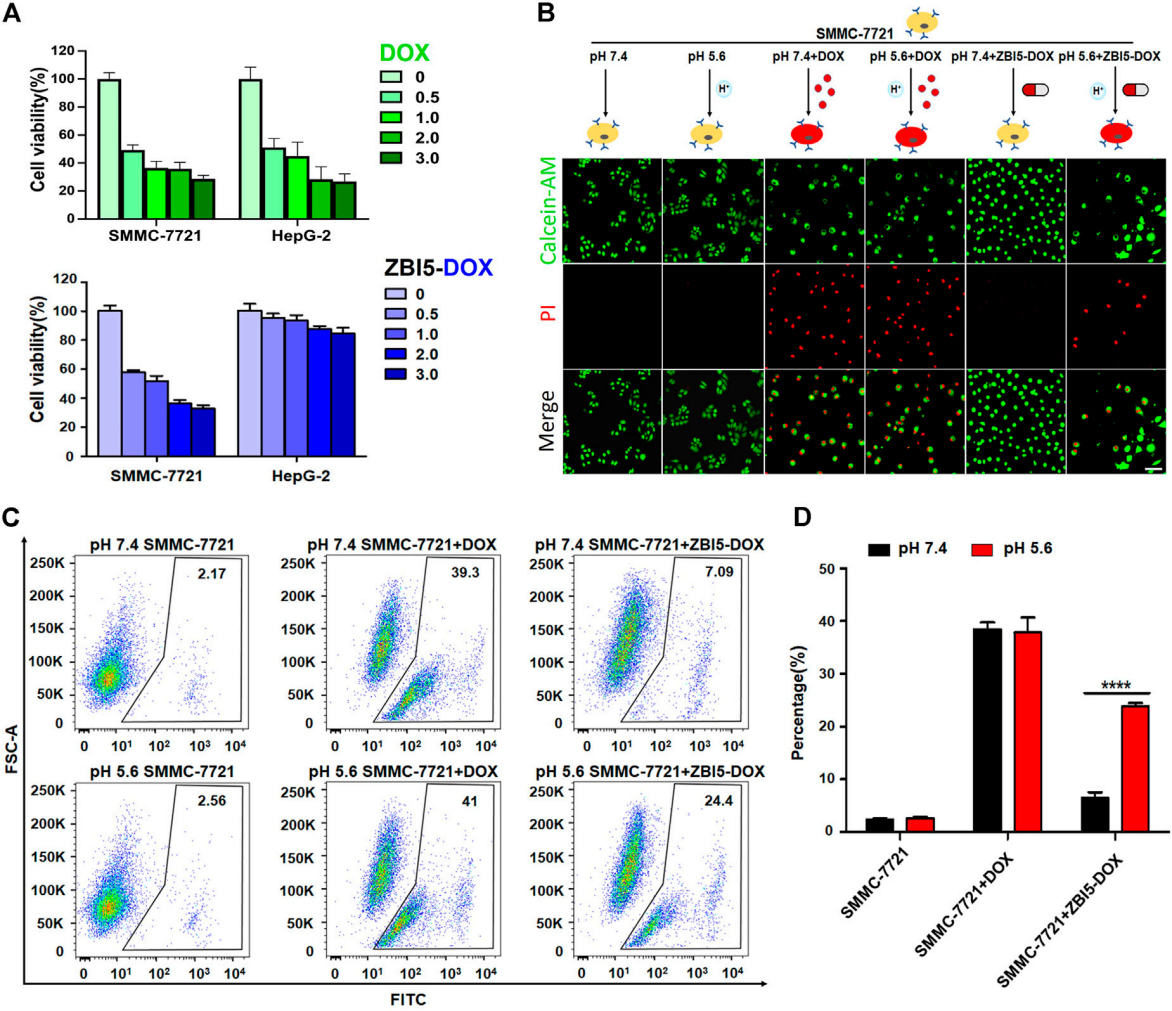
Selective cytotoxicity of ZBI5-DOX. **(A)** MTT assays of cell viability of SMMC-7721 and HepG-2 cells treated with different concentration (0, 0.5, 1.0, 2.0 and 3.0 μM) of DOX or ZBI5-DOX in pH 5.6. **(B)** LSCM images of SMMC-7721 cells treated with free DOX and ZBI5-DOX in pH 5.6 and 7.4. Untreated SMMC-7721 cells were used as controls. Calcein-AM-labeled living cells were indicated as green, and PI-labeled dead cells were indicated as red. **(C)** Flow cytometry assays of free DOX and ZBI5-DOX-induced apoptosis of SMMC-7721 cells via caspase-3 detection. Untreated cells were used as controls. The black box indicates the positive area. **(D)** Quantitative analysis of the percentage of cells located in the positive areas. The data was presented as means ± SD, and the asterisk indicates a significant difference (****p < 0.0001).

To quantitatively evaluate the selective cytotoxicity of the smart DNA nanocapsule, MTT experiments were implemented to detect the cell viability. Before evaluation, the size, zeta potential, and the biocompatibility of ZBI5 vehicles without DOX were investigated. As seen in Supplementary Figure S20, the size obtained with dynamic light scattering of the DNA nanocapsule was about 21 nm and the zeta potential was -13.1 mV. Supplementary Figure S21 indicated that both target SMMC-7721 and control HepG-2 cells displayed no obvious cytotoxicity to different concentration of ZBI5 in pH 5.6 and pH 7.4, meaning great biocompatibility of ZBI5 vehicles for biomedical applications. When treated with ZBI5-DOX, target cells showed decreased cell viability with increased concentration of ZBI5-DOX, similar to incubated with the free DOX. But control cells showed high cell viability to different concentration of ZBI5-DOX compared with the free DOX (Figure 7A). Besides, the toxicity of ZB5-DOX, ZI13-DOX and ZBI5-DOX were evaluated, as displayed in Supplementary Figure S22, only ZBI5-DOX showed specific cytotoxicity to target cells in pH 5.6 compared with the controls. These results proved that the DNA nanocapsule had excellent selective cytotoxicity to target cells. To visualize the selective cytotoxicity of ZBI5-DOX, the cells were stained with Calcein-AM and PI dyes for confocal imaging. Calcein-AM was able to stain living cells, and PI was able to stain dead cells. As shown in Figure 7B, SMMC-7721 cells treated with ZBI5-DOX in acidic conditions showed reduced green fluorescence and obvious red fluorescence compared to SMMC-7721 cells treated with ZBI5-DOX in pH 7.4, indicating selective target cell killing of ZBI5-DOX in acidic conditions. In contrast, free DOX showed nonselective killing of both target and control HepG-2 cells in pH 5.6 and 7.4. Besides, no red fluorescence and changed green fluorescence were observed in control cells treated with ZBI5-DOX in pH 5.6 and 7.4, further verified selective cytotoxicity of ZBI5-DOX (Supplementary Figure S23).

Caspase-3 is a vital effector protease in the process of apoptosis, which promoted the occurrence of apoptosis (Porter and Janicke, 1999). Therefore, the activity of caspase-3 was detected to evaluate ZBI5-DOX-induced apoptosis. Dye-labeled caspase-3 substrates were used as indicators, of which fluorescence could be activated by caspase-3. As demonstrated in Figure 7C, for untreated target SMMC-7721 cells in pH 5.6 and 7.4, we observed only a small number of events in positive areas, for target cells treated with free DOX in pH 5.6 and 7.4, 39.3% and 41% of cells were respectively appeared in positive areas due to nonselective killing of free DOX. In contrast, we observed 24.4% of cells showed in positive area for ZBI5-DOX-treated target cells in pH 5.6, but 7.09% for ZBI5-DOX-treated target cells in pH 7.4, indicating specific killing of ZBI5-DOX in acidic conditions (Figure 7D). In addition, we observed no distinctive events in positive areas for control HepG-2 cells (Supplementary Figure S24). These results showed that ZBI5-DOX could induce specific apoptosis of target cells, which contributed to the selective cytotoxicity of the DNA nanocapsule.

### *In vitro* inhibition of cell migration and invasion

**FIGURE 8:**
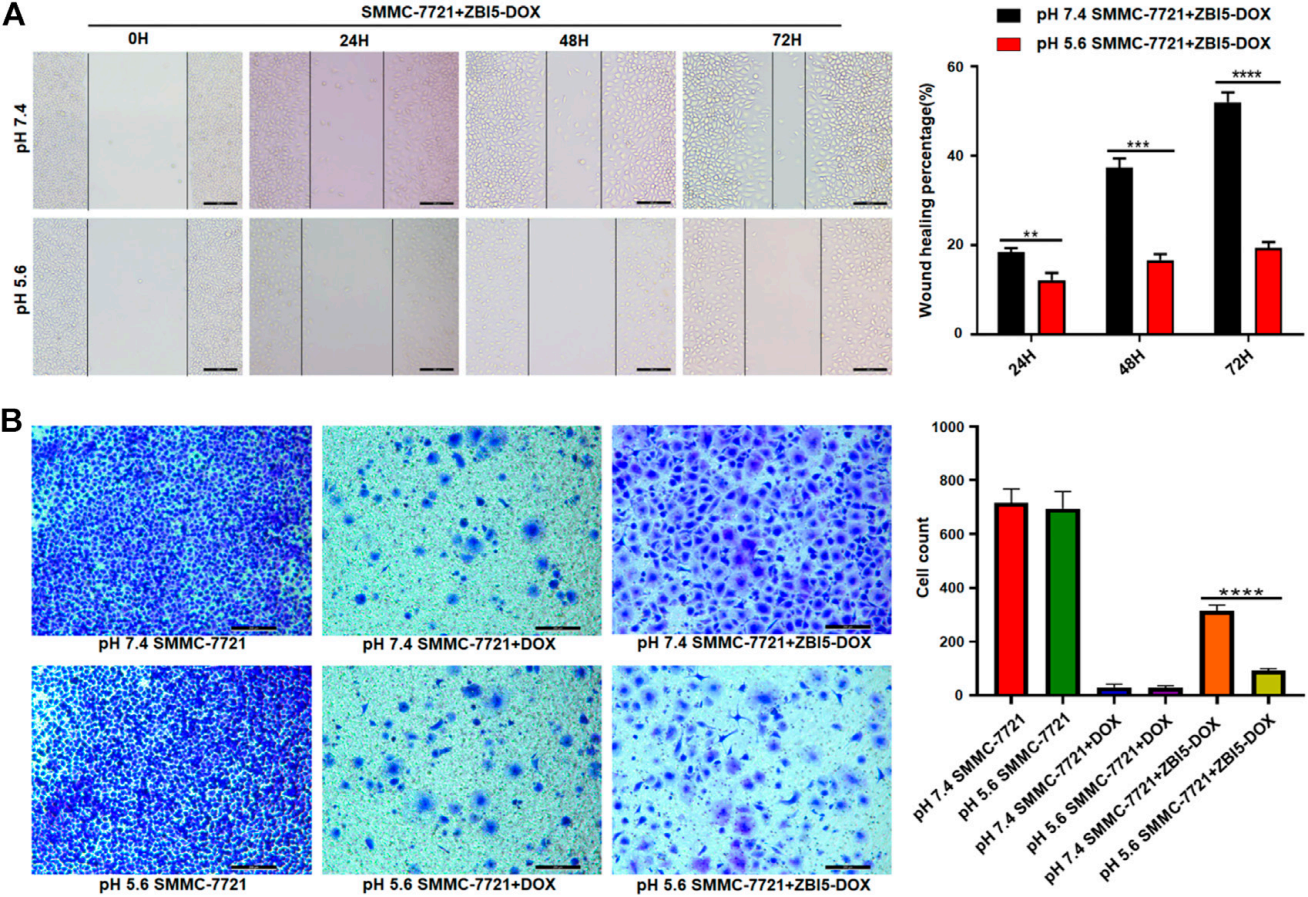
Investigation of cell migration and invasion induced by the DNA nanocapsule. **(A)** Wound healing assays of target SMMC-7721 cells treated with ZBI5-DOX in pH 5.6 and 7.4. **(B)** Transwell invasion assays of SMMC-7721 cells treated with free DOX and ZBI5-DOX in pH 5.6 and 7.4. Untreated cells were used as controls. Quantitative analysis was performed, the data was presented as means ± SD, and the asterisk indicates a significant difference (**p < 0.01, ***p < 0.001, ****p < 0.0001).

To investigate the effects of the DNA nanocapsule on the cell migration and invasion, wound healing and Transwell invasion assays were performed. Wound healing assay was used to evaluate collective cell migration and Transwell invasion assay was used to assess cell invasion behaviors *in vitro*. The results of wound healing assays showed specific cell migration inhibition of ZBI5-DOX treated target SMMC-7721 cells in acidic conditions (Figure 8A), but nonselective cell migration inhibition of free DOX to target cells and control HepG-2 cells, and no cell migration inhibition ZBI5-DOX to HepG-2 cells (Supplementary Figures S25,S26). Furthermore, Transwell invasion assays showed that the invaded cell counts of ZBI5-DOX treated target cells in acidic conditions were distinctly less than that of target cells in pH 7.4 (Figure 8B), while ZBI5-DOX treated control cells showed large number of cell counts in pH 5.6 and 7.4 (Supplementary Figure S27). These results indicated that ZBI5-DOX could selectively inhibit target cell migration and invasion in acidic conditions, suggesting great potential of the DNA nanocapsule for specific inhibition of tumor cell behaviors.

## Conclusion

In summary, we have designed and constructed a smart sequentially triggered DNA nanocapsule for targeted drug delivery based on an acidic pH-responsive i-motif DNA and a tumor cell-specific aptamer. By using this smart DNA nanocapsule, DOX could be precisely delivered to target SMMC-7721 tumor cells in acidic conditions, and selective *in vitro* cytotoxicity of the DNA nanocapsule to target cells was successfully achieved. Moreover, the DNA nanocapsule-induced target cell apoptosis was verified by caspase-3 detection, and specific inhibition of target cell migration and invasion were also verified *via* wound healing and Transwell invasion assays. Theoretically, the proposed strategy is available to deliver drug to different tumor cells at various acidic pH just by replacing the i-motif and aptamer sequences. Therefore, this sequentially triggered strategy holds great potential in developing new therapeutics for targeted cancer treatments.

## Data Availability

The original contributions presented in the study are included in the article/Supplementary Material, further inquiries can be directed to the corresponding authors.
