# Supplementary material for: A sequentially triggered DNA nanocapsule for targeted drug delivery based on pH-responsive i-motif and tumor cell-specific aptamer
Source: Front Bioeng Biotechnol. 2022 Aug 25;10:965337. doi: 10.3389/fbioe.2022.965337 (PMC9453301; doi:10.3389/fbioe.2022.965337)
Supplement: Supplementary file 1 [file DataSheet1.docx]

**Supporting Information**

**A sequentially triggered DNA nanocapsule for targeted drug delivery based on pH-responsive i-motif and tumor cell-specific aptamer**

*Baoyin Yuan*^1^ *^†^, Yanan Xi^1 †^, Cuihua Qi^1^, Mingzhu Zhao^1^, Xiaoyan Zhu^1^* and Jinlu Tang^1^**

^1^ School of Basic Medical Sciences, Zhengzhou University, Zhengzhou, Henan Province 450001, PR China

^†^ These authors make equal contribution to the paper.

* Corresponding Authors. E-mail: tangjl@zzu.edu.cn; zhxy@zzu.edu.cn.

**Table S1**. DNA sequences used in this work.

| Name | Sequence (5’to3’) |
| --- | --- |
| B1 | CAGAGCAG-BHQ2 |
| B2 | CCAGAGCAG-BHQ2 |
| B3 | CCCAGAGCAG-BHQ2 |
| B4 | ACCCAGAGCAG-BHQ2 |
| B5 | GACCCAGAGCAG-BHQ2 |
| B6 | GGACCCAGAGCAG-BHQ2 |
| B7 | CGGACCCAGAGCAG-BHQ2 |
| B8 | ACGGACCCAGAGCAG-BHQ2 |
| Z11-L | ACGGACTACCTGACG-(Cy5)- CTGCTCTGGGTCCGTCAGGTTGAGCTGAAGATCGTAC CGTGAAGTCCGT |
| I9 | CCCCCCTCCCCCC-(BHQ1)-ACGGACTTCTTTTCCCCCCTCCCCCC |
| I10 | CCCCCCTCCCCCC-(BHQ1)-ACGGACTTCATTTCCCCCCTCCCCCC |
| I11 | CCCCCCTCCCCCC-(BHQ1)-ACGGACTTCACTTCCCCCCTCCCCCC |
| I12 | CCCCCCTCCCCCC-(BHQ1)-ACGGACTTCACGTCCCCCCTCCCCCC |
| I13 | CCCCCCTCCCCCC-(BHQ1)-ACGGACTTCACGGATCCCCCCTCCCCCC |
| cI13 | TTTTTTTTTTTTT-(BHQ1)-ACGGACTTCACGGATTTTTTTTTTTTTT |
| I14 | CCCCCCTCCCCCC-(BHQ1)-ACGGACTTCACGGTTCCCCCCTCCCCCC |
| Z11-I | ACGGACTACCTGACGTCTGCTCTGGGTCCGTCAGGTTGAGCTGAAGATCGTACCGTGAAGTCCGT-Alexa Fluor 488 |


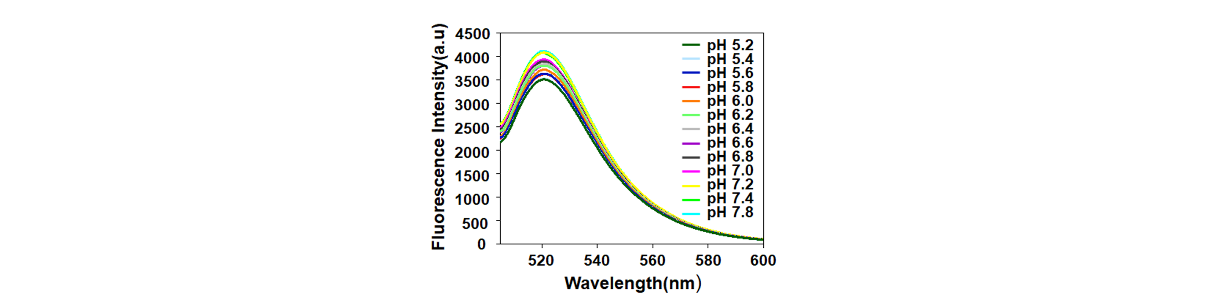


**FIGURE S1 |** Fluorescence spectra of Alexa 488 labeled Z11 in pH 5.2-7.8 at 25 ℃. The results indicated that the Alexa 488 was pH-insensitive fluorescent group. The concentration of Z11 probes was 100 nM.


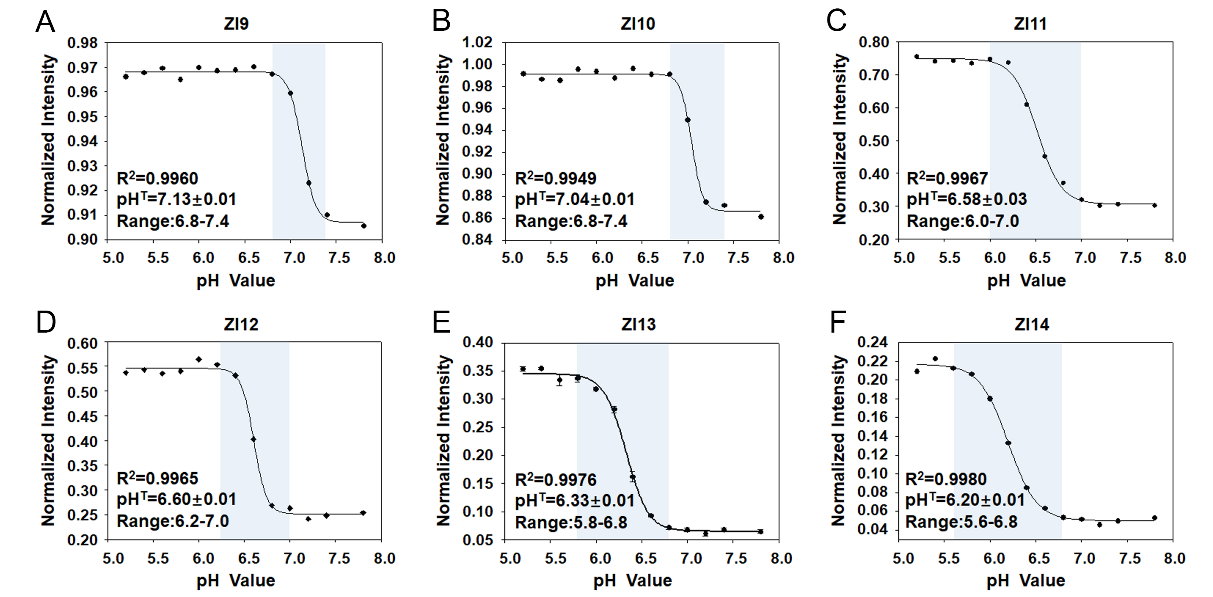


**FIGURE S2 |** The fluorescence profile of ZI9-14 (100 nM) in PBS at 25 ℃. The normalized intensity represents the ratio of fluorescence intensity of ZI9-14 to Alexa 488-labeled Z11. pH^T^ indicates responsive pH median.


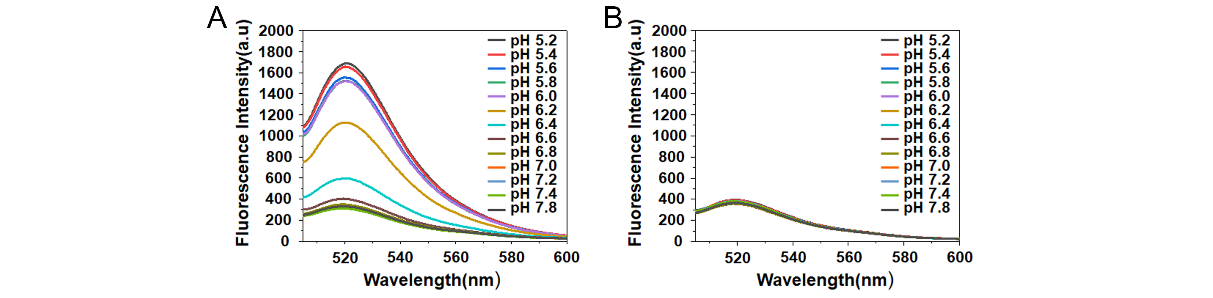


**FIGURE S3 |** Fluorescence spectra of (A) ZI13 and (B) cZI13 in the pH range from 5.2 to 7.8. The concentration of probes was 100 nM, respectively.


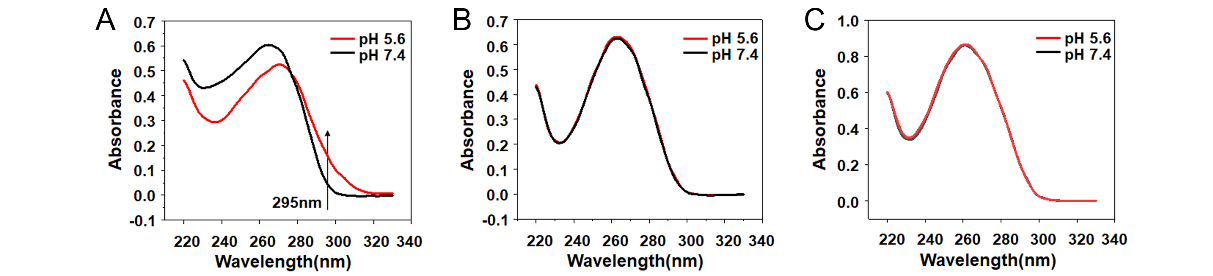


**FIGURE S4 |** UV absorbance spectrum of I13 (A), cI13 (B) and cZI13 (C) at pH 5.6 and 7.4. The arrow indicates absorbance at 295 nm.


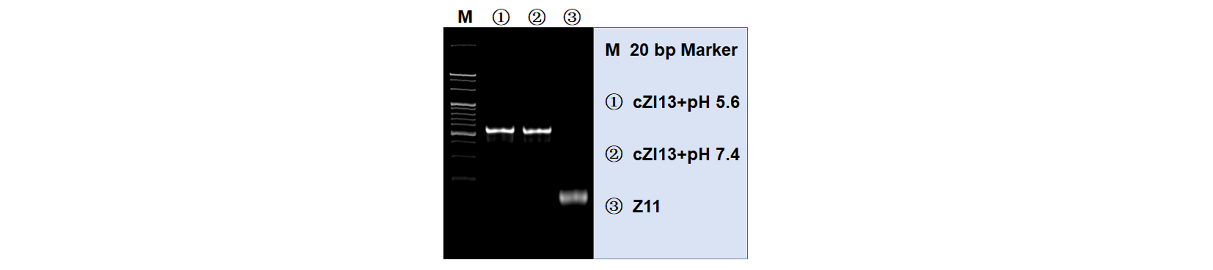


**FIGURE S5 |** PAGE images of cZI13 at pH 5.6 (lane 1), cZI13 at pH 7.4 (lane 2), and Z11 (lane 3).


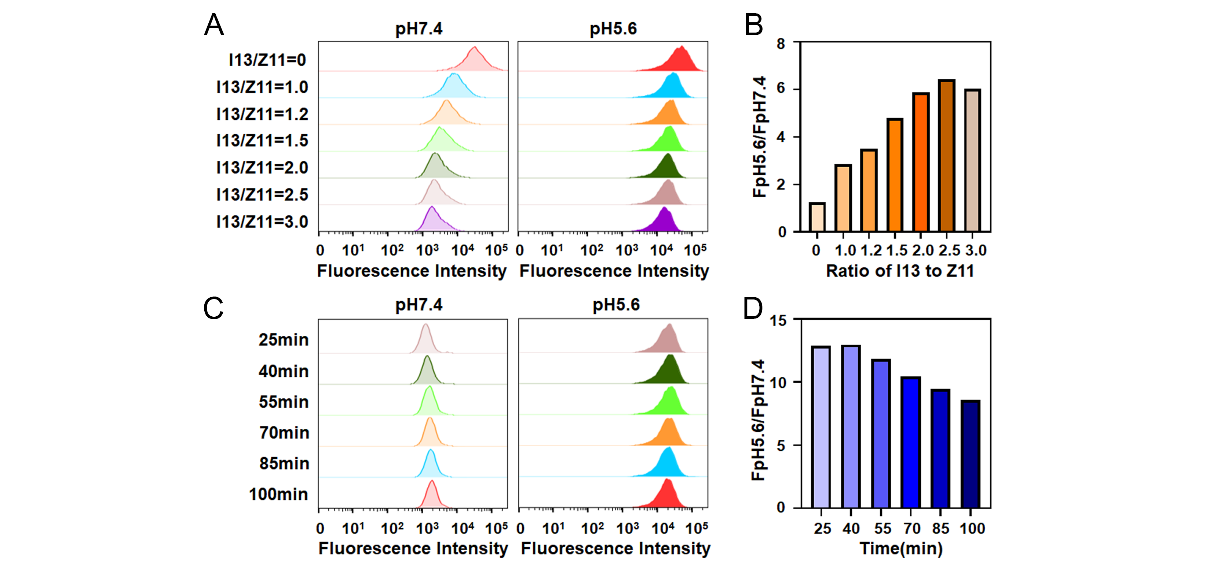


**FIGURE S6 |** Optimization of the ratio I13 to Z11 and incubation time. (A) Flow cytometry assays of ZI13 binding to target SMMC-7721 cells with different ratio I13 to Z11 (0, 1.0, 1.2, 1.5, 2.0, 2.5 and 3.0) at pH 7.4 and pH 5.6. (B) SBR of ZI13 with different ratio I13 to Z11. (C) Flow cytometry assays of ZI13 binding to target SMMC-7721 cells with different incubation time (25, 40, 55, 70, 85, and 100 min) at pH 7.4 and pH 5.6. (D) SBR of ZI13 with different incubation time. SBR represents fluorescence intensity at pH 5.6 to fluorescence intensity at pH 7.4.


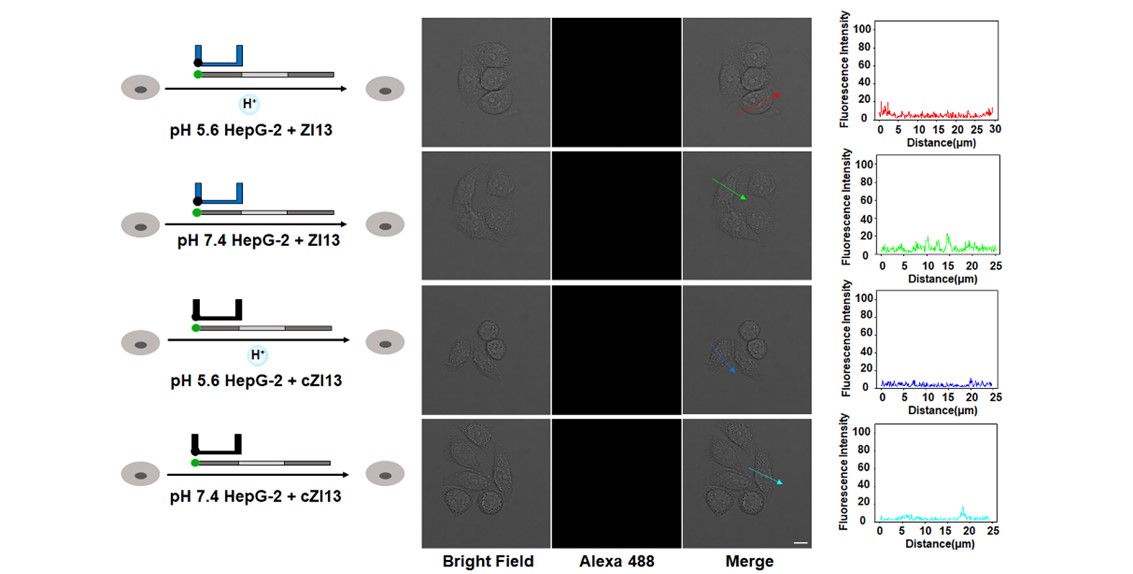


**FIGURE S7 |** LSCM images of HepG-2 cells incubated with ZI13 and cZI13 at pH 5.6 and pH 7.4. The fluorescence intensity indicated by the arrows was listed right. The fluorescence signal was collected by a 100× objective (fluorescence channel: EX 488 nm, EM 525 nm long-pass).


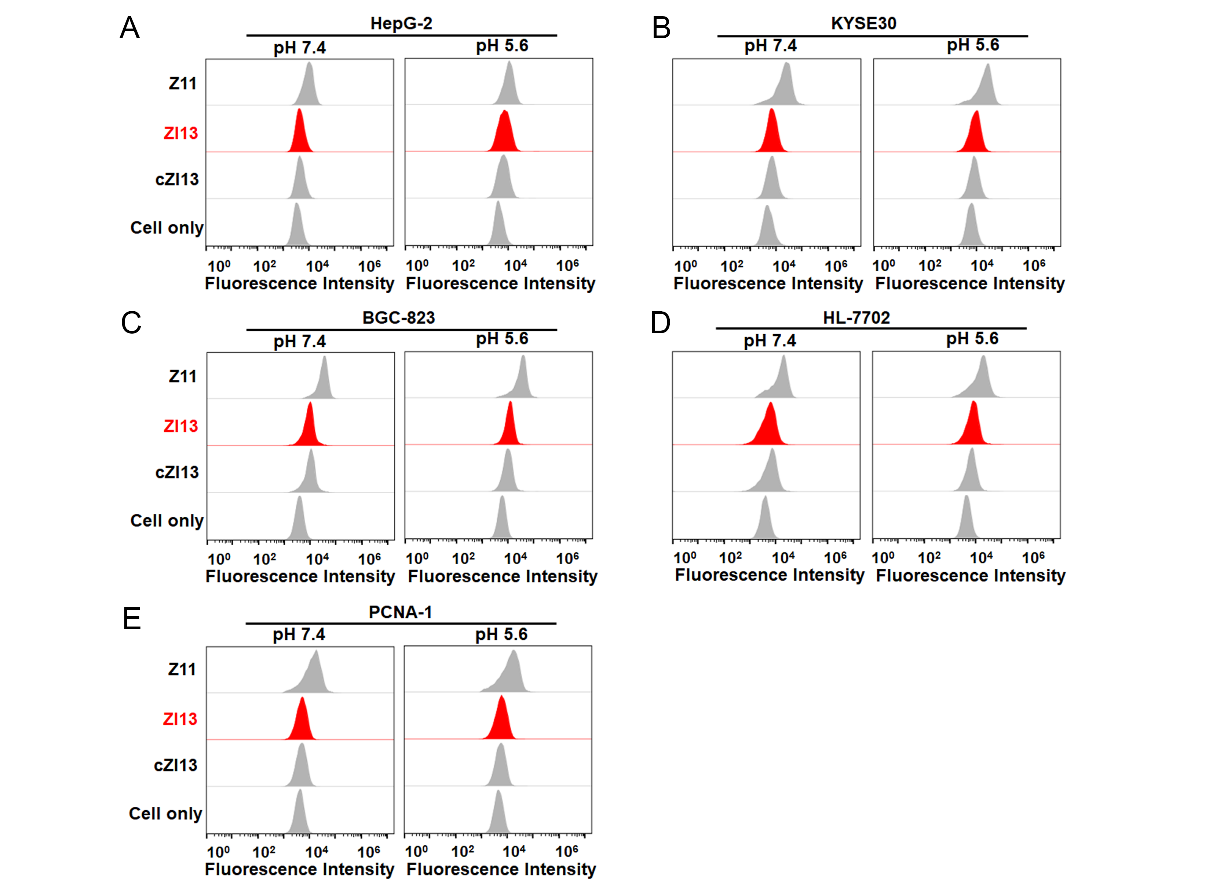


**FIGURE S8 |** Flow cytometry assays of binding of ZI13 to five control cell lines at pH 7.4 and pH 5.6. (A) Hepatocellular carcinoma HepG-2 cells, (B) esophageal carcinoma KYSE30 cells, (C) gastric carcinoma BGC-823 cells, (D) hepatocyte HL-7702 cells, and (E) pancreatic carcinoma PCNA-1 cells. Z11 was used as positive controls and cZI13 was used as negative controls. Alexa 488 was collected as fluorescence signal.


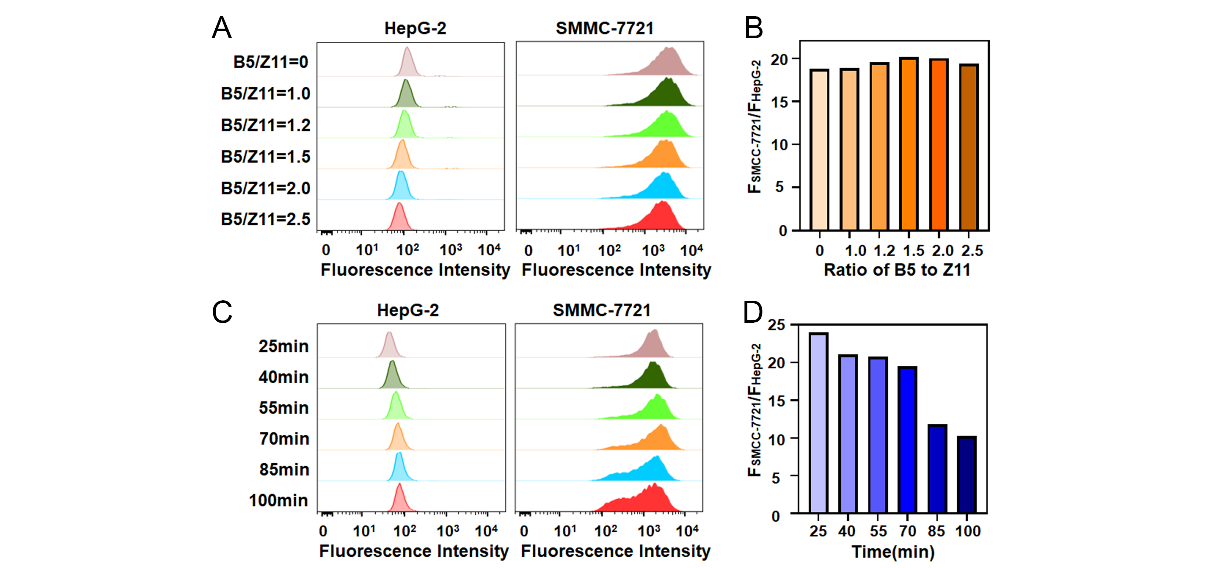


**FIGURE S9 |** Optimization of the ratio of B5 to Z11 and incubation time. (A) Flow cytometry assays of ZB5 binding to target SMMC-7721 and control HepG-2 cells with different ratio of B5 to Z11 (0, 1.0, 1.2, 1.5, 2.0 and 2.5). (B) SBR of ZB5 with different ratio of B5 to Z11. (C) Flow cytometry assays of ZB5 binding to target SMMC-7721 and control HepG-2 cells with different incubation time (25, 40, 55, 70, 85 and 100 min). (D) SBR of ZB5 with different incubation time. SBR represents fluorescence intensity of target SMMC-7721 cells to fluorescence intensity of control HepG-2 cells.


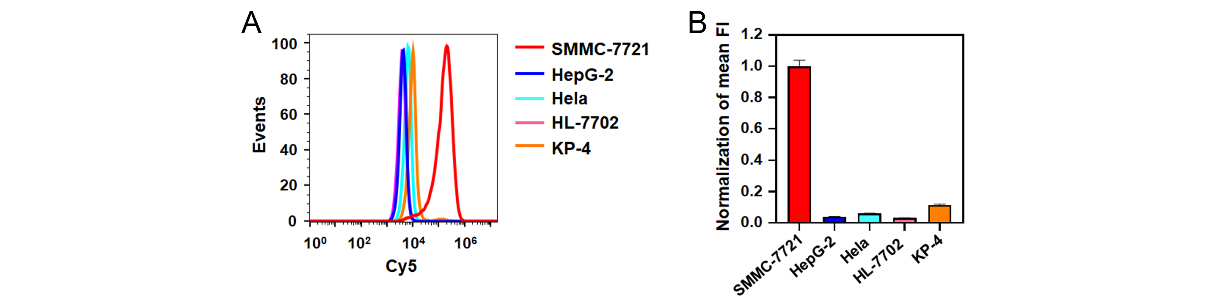


**FIGURE S10 |** Evaluation of binding specificity of ZB5. (A) Flow cytometry assays of target SMMC-7721 cells, control HepG-2 cells, Hela cells, HL-7702 cells and KP-4 cells incubated with ZB5. (B) Corresponding mean fluorescence intensity (FI) of target cells and control cells incubated with ZB5.


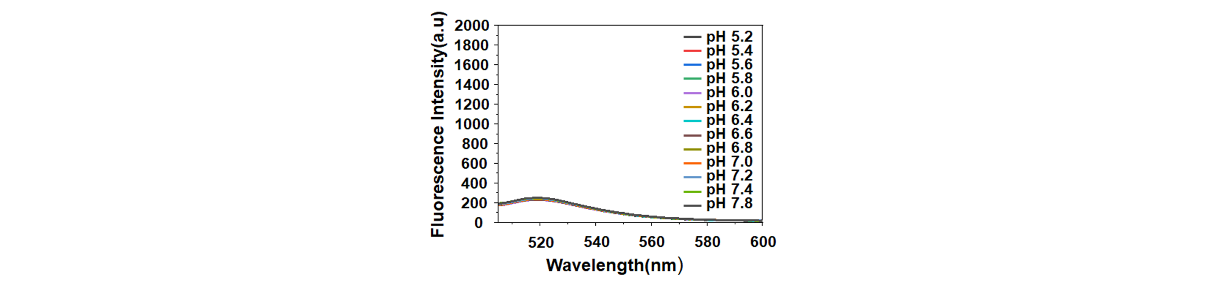


**FIGURE S11 |** Fluorescence spectrum of cZBI5 control in pH 5.2-7.8. The concentration of cZBI5 control was 100 nM.


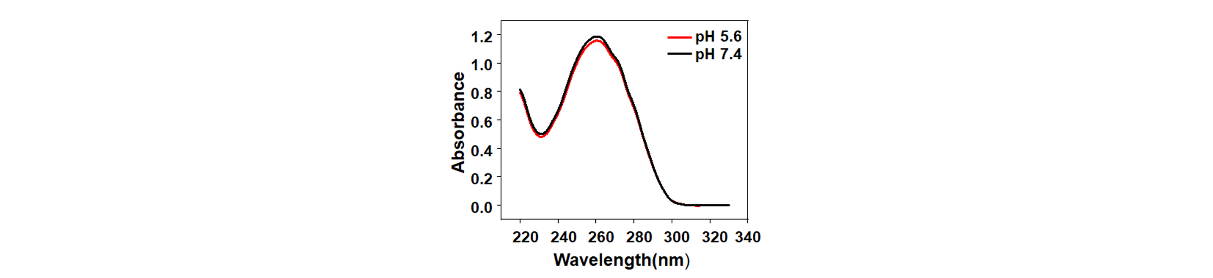


**FIGURE S12 |** UV absorbance spectrum of cZBI5 control at pH 5.6 and pH 7.4.


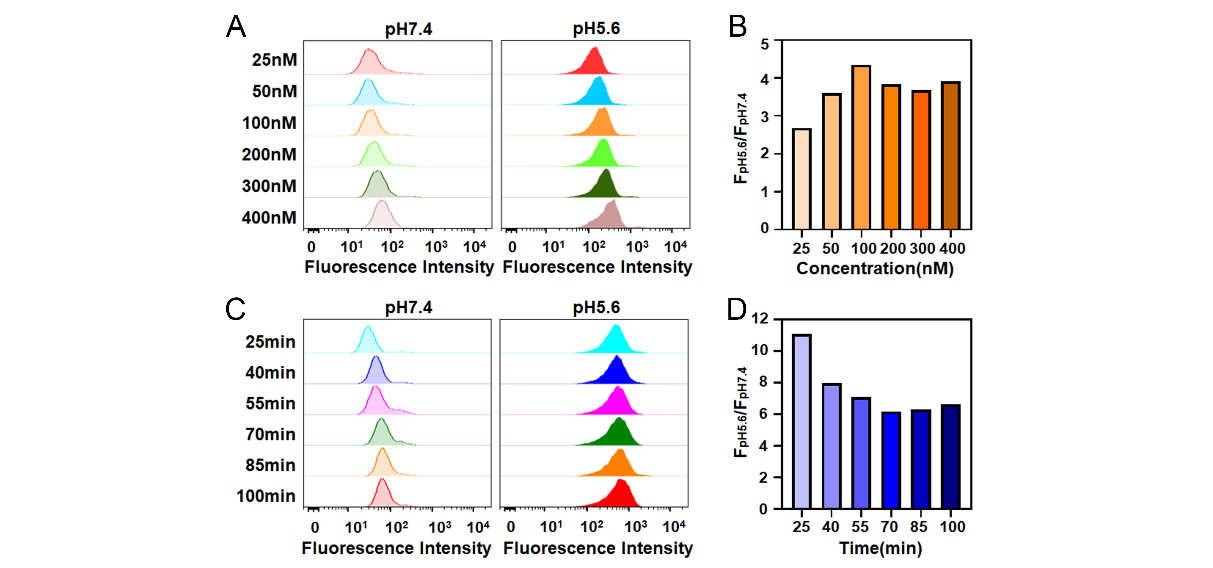


**FIGURE S13 |** Optimization of the incubation concentration and time. (A) Flow cytometry assays of different concentration of ZBI5 (25, 50, 100, 200, 300 and 400 nM) binding to target SMMC-7721 cells in pH 7.4 and pH5.6. (B) SBR of different concentration of ZBI5. (C) Flow cytometry assays of ZBI5 binding to target SMMC-7721 cells with different incubation time (25, 40, 55, 70, 85 and 100 min). (D) SBR of ZBI5 with different incubation time. SBR represents fluorescence intensity in pH5.6 to fluorescence intensity in pH7.4.


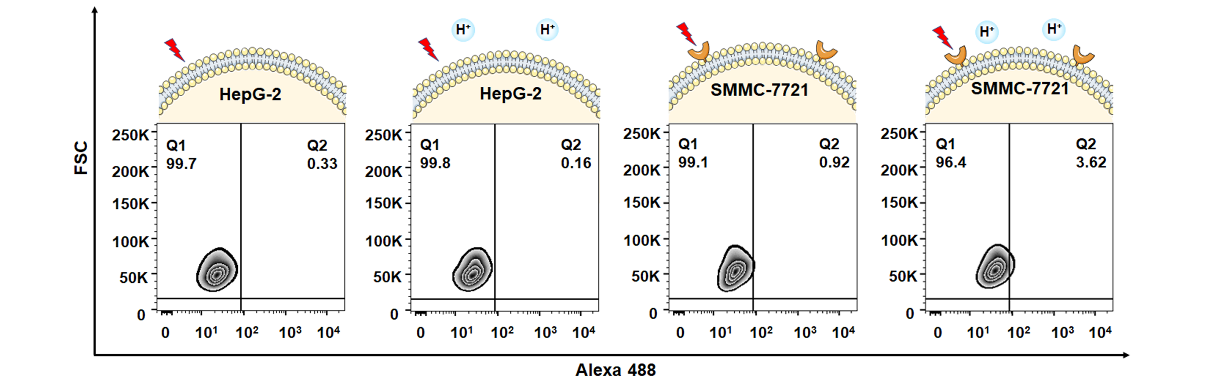


**FIGURE S14 |** Flow cytometry assays of recognition of cZBI5 to target SMMC-7721 and control HepG-2 cells in acidic (pH 5.6) and non-acidic (pH 7.4) conditions.


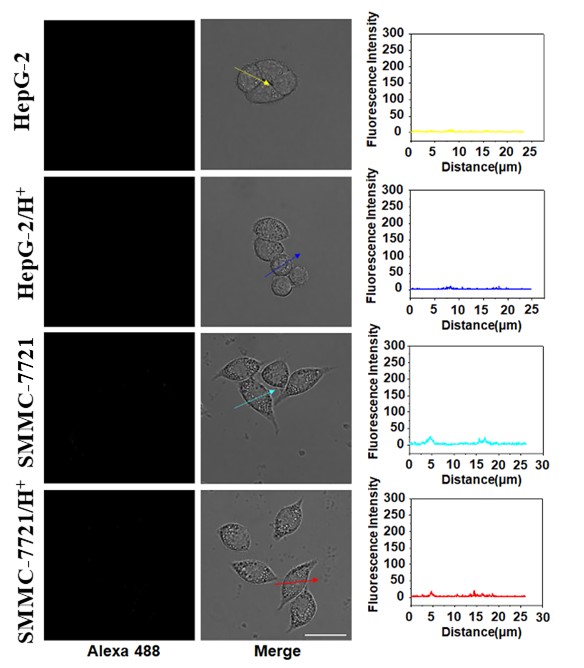


**FIGURE S15 |** LSCM images of target SMMC-7721 cells and control HepG-2 cells incubated with cZBI5 in pH 5.6 and pH 7.4. The fluorescence intensity indicated by the arrows was listed right. The fluorescence signal was collected by a 100× objective (fluorescence channel: EX 488 nm, EM 525 nm long-pass).


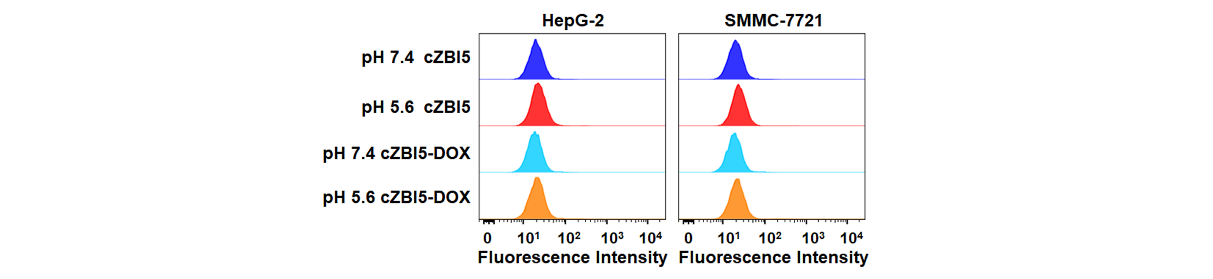


**FIGURE S16 |** Flow cytometry assays of target SMMC-7721 cells and control HepG-2 cells incubated with cZBI5 and cZBI5-DOX in pH 5.6 and pH 7.4, respectively.


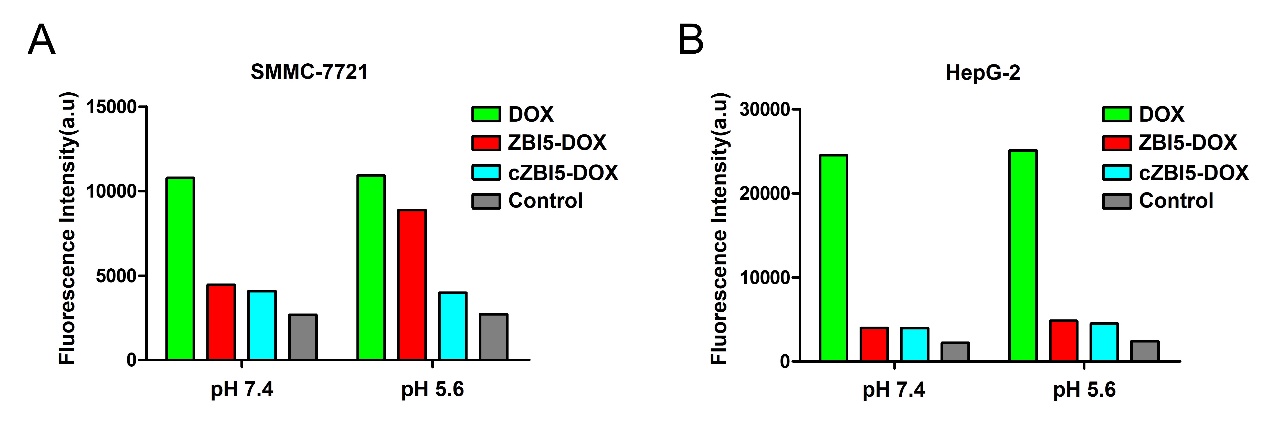


**FIGURE S17 |** The corresponding histogram of the DOX fluorescence of target SMMC-7721 cells (A) and control HepG-2 cells (B) incubated with free DOX, cZBI5-DOX and ZBI5-DOX in pH 5.6 and 7.4.


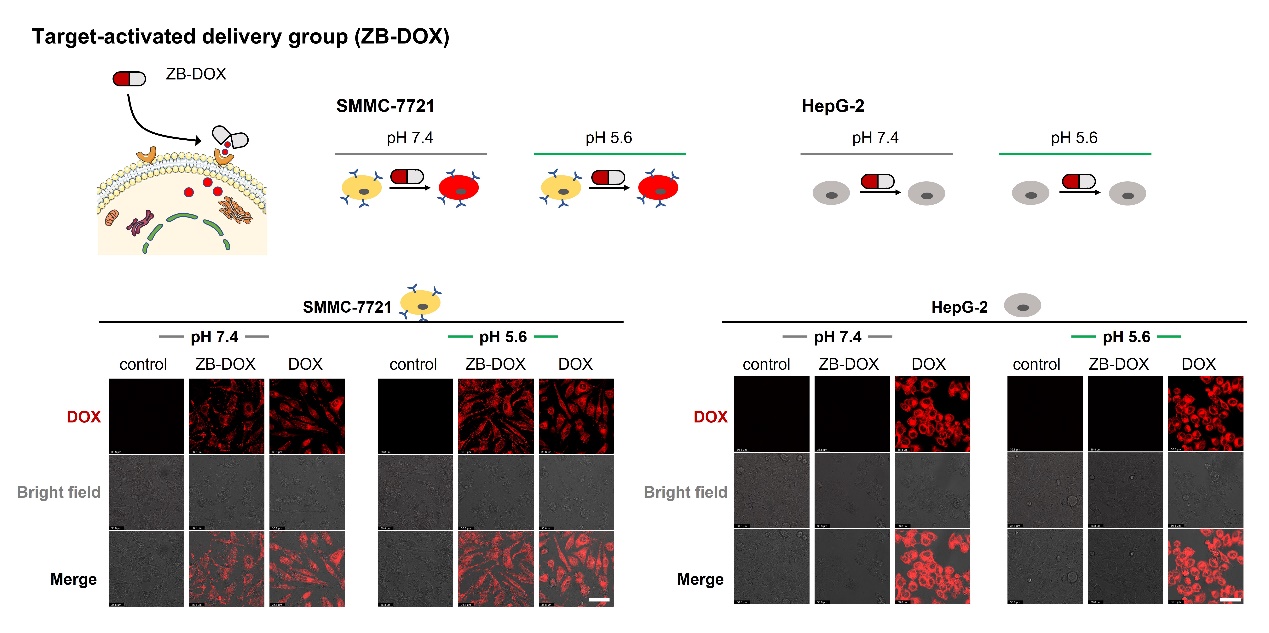


**FIGURE S18 |** Confocal imaging of target SMMC-7721 cells and control HepG-2 cells incubated with free DOX, ZB-DOX in pH 5.6 and pH 7.4, respectively. The scale bar is 50 μm.


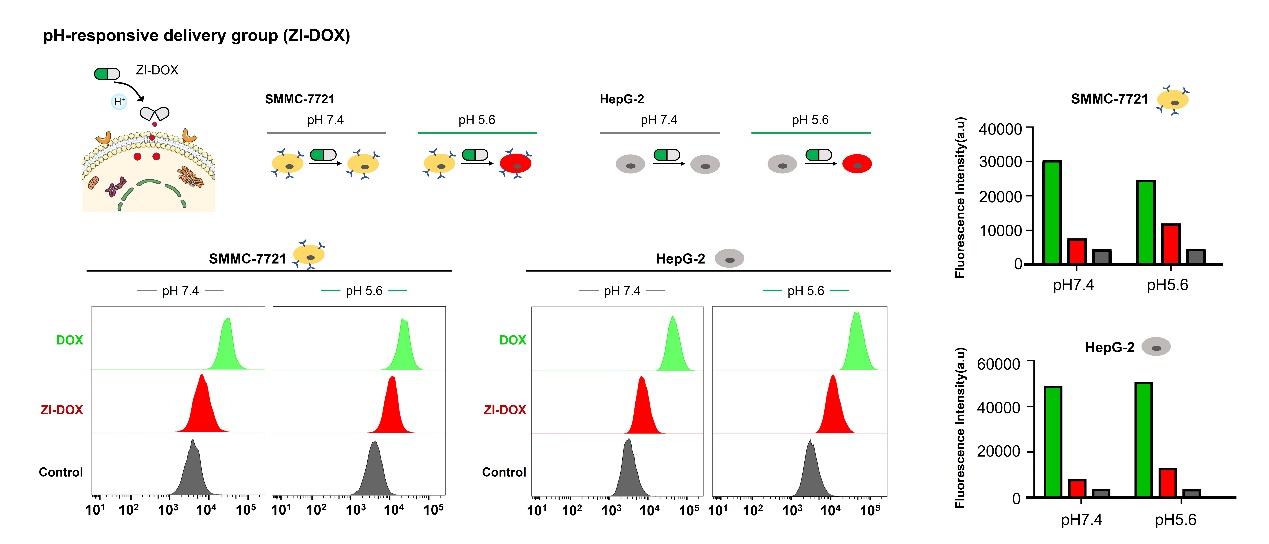


**FIGURE S19 |** Flow cytometry assays and the corresponding histogram of the DOX fluorescence of target SMMC-7721 cells and control HepG-2 cells incubated with free DOX, ZI-DOX in pH 5.6 and pH 7.4, respectively.


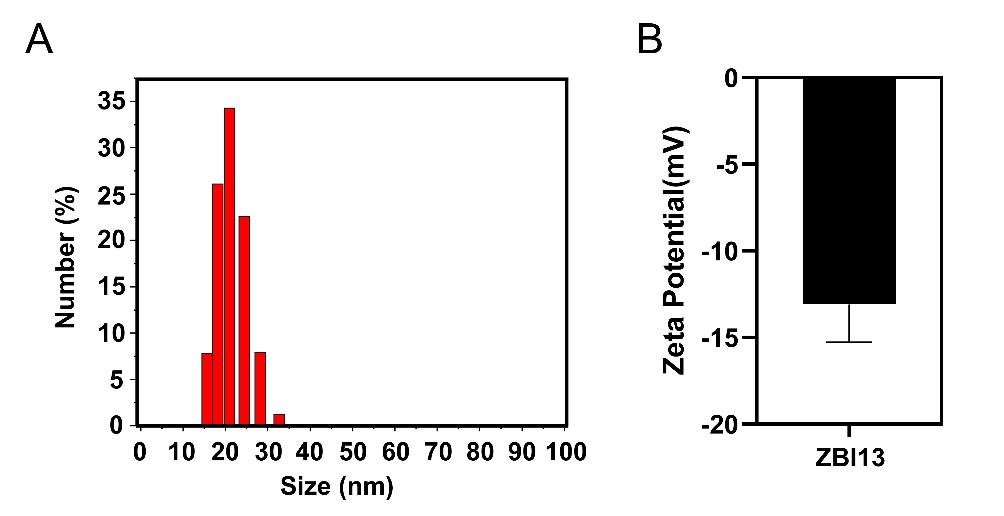


**FIGURE S20 |** Characterization of ZBI13 nanocapsule. (A) Size distribution, (B) zeta potential.


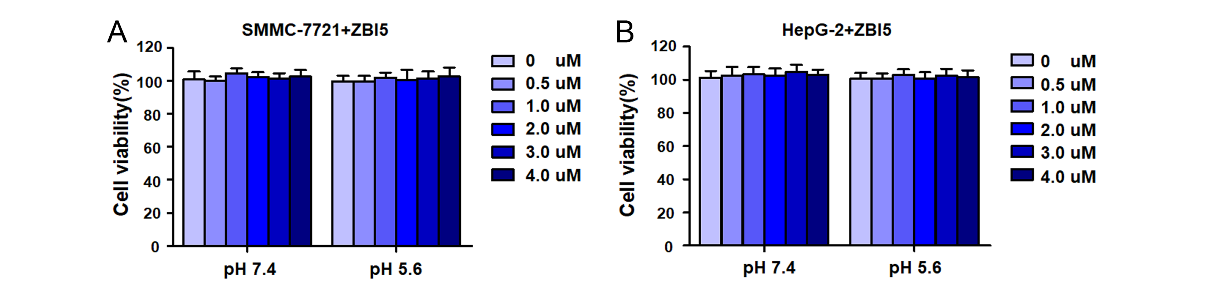


**FIGURE S21 |** MTT assays of cell viability of SMMC-7721 (A) and HepG-2 (B) cells treated with different concentration (0, 0.5, 1.0, 2.0, 3.0 and 4.0 μM) of ZBI5 in pH 5.6 and pH 7.4.


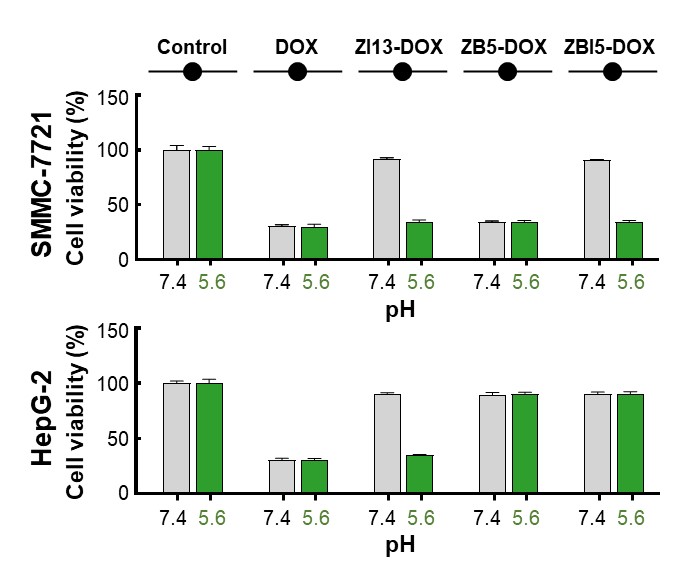


**FIGURE S22 |** MTT assays of cell viability of SMMC-7721 and HepG-2 cells treated with 3μM of DOX, ZI13-DOX, ZB5-DOX and ZBI5-DOX in pH 5.6 and pH 7.4. Untreated cells were used as controls.


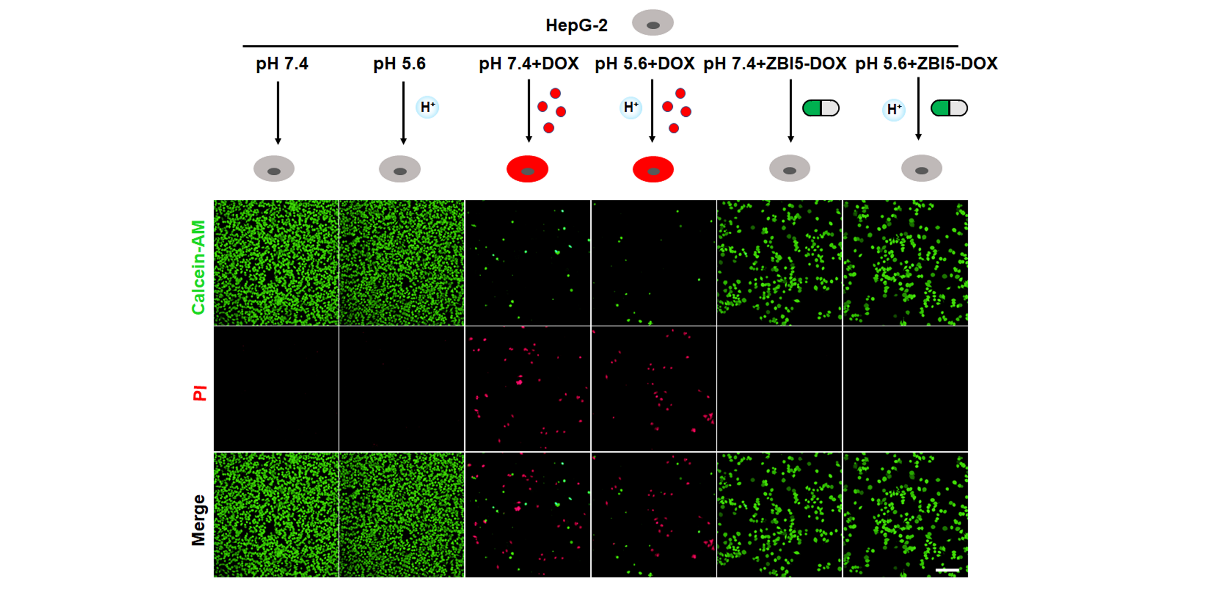


**FIGURE S23 |** LSCM images of HepG-2 cells treated with free DOX and ZBI5-DOX in pH 5.6 and pH 7.4. Untreated SMMC-7721 cells were used as controls. Calcein-AM-labeled living cells were indicated as green, and PI-labeled dead cells were indicated as red. The scale bar is 100 μm.


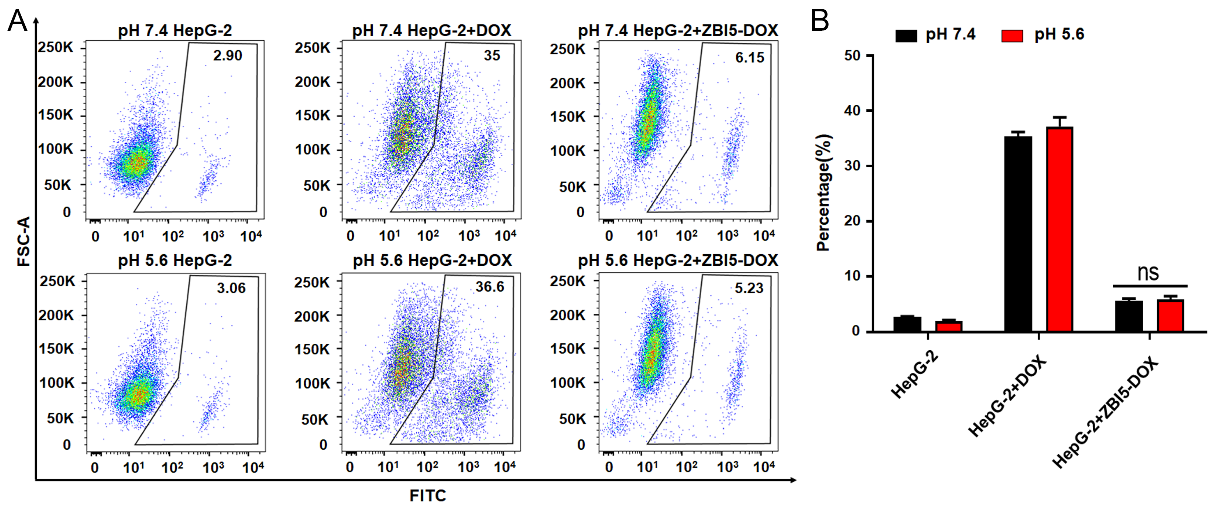


**FIGURE S24 |** Analysis of ZBI5-DOX-induced apoptosis of HepG-2 cells through caspase-3 detection. (A) Flow cytometry assays of free DOX and ZBI5-DOX-induced apoptosis of HepG-2 cells via caspase-3 detection. Untreated cells were used as controls. The black box indicates the positive area. (B) Quantitative analysis of the percentage of cells located in the positive areas. The data was presented as means ± SD, and ns indicates no significance.


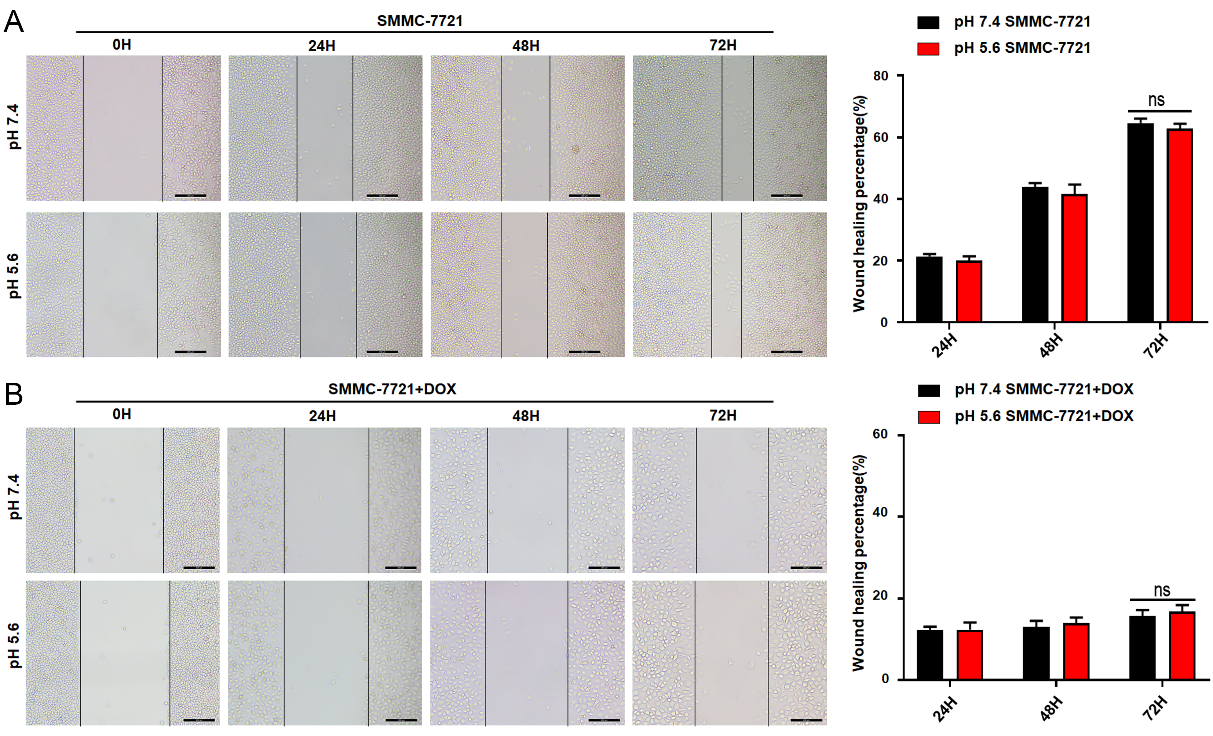


**FIGURE S25 |** Wound healing assays of untreated SMMC-7721 cells (A) and SMMC-7721 cells treated with free DOX (B). The asterisks indicate that the cell migration in pH 5.6 were significantly lower than that in pH 7.4. ns indicates no significance.


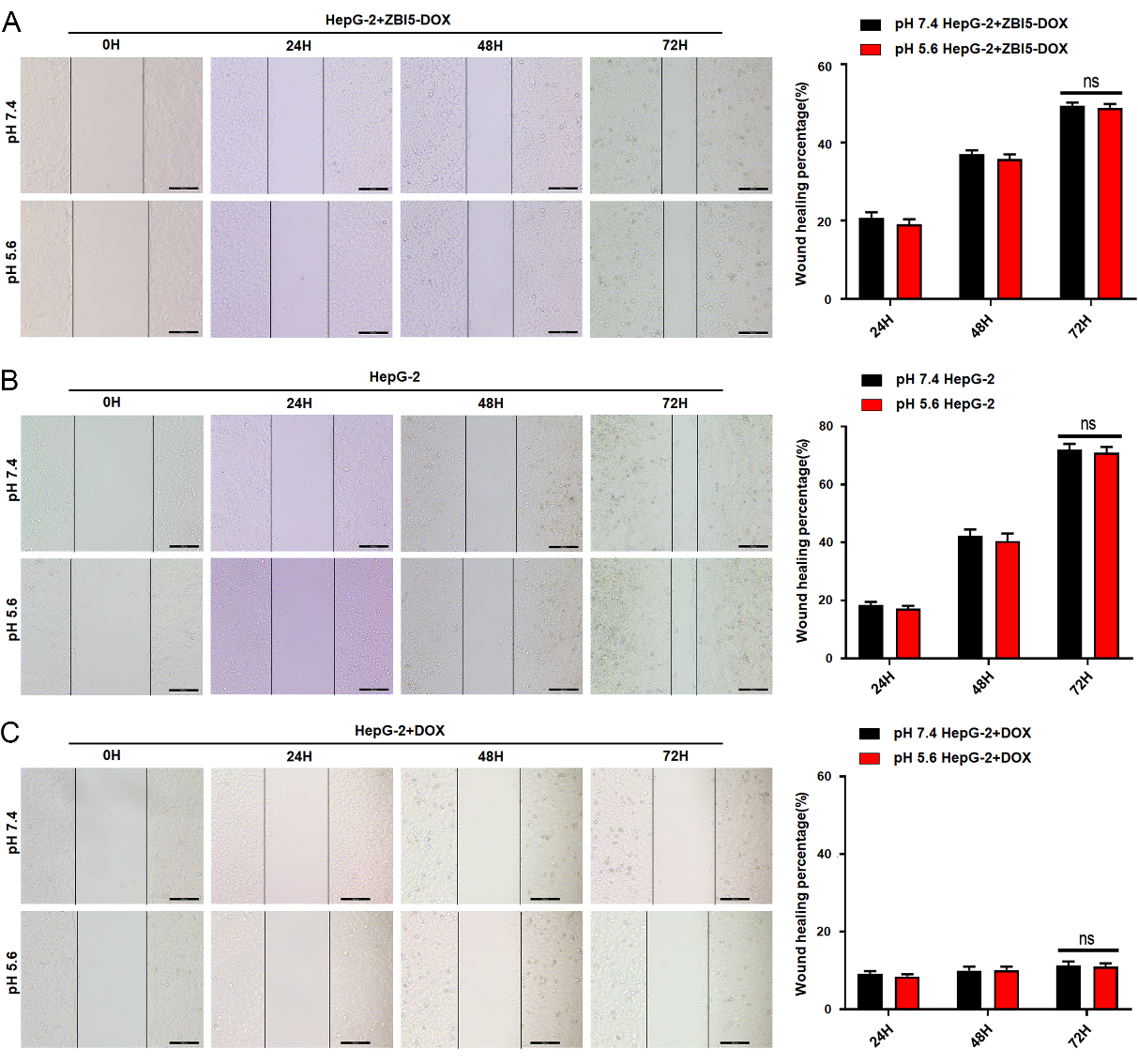


**FIGURE 26 |** Investigation of cell migration induced by the DNA nanocapsule. Wound healing assays of control HepG-2 cells treated with ZBI5-DOX (A), untreated HepG-2 cells (B), and HepG-2 cells treated with free DOX (C) in pH 5.6 and 7.4. The asterisks indicate that nonselective cell migration inhibition of ZBI5-DOX and free DOX treated control cells at different pH values.


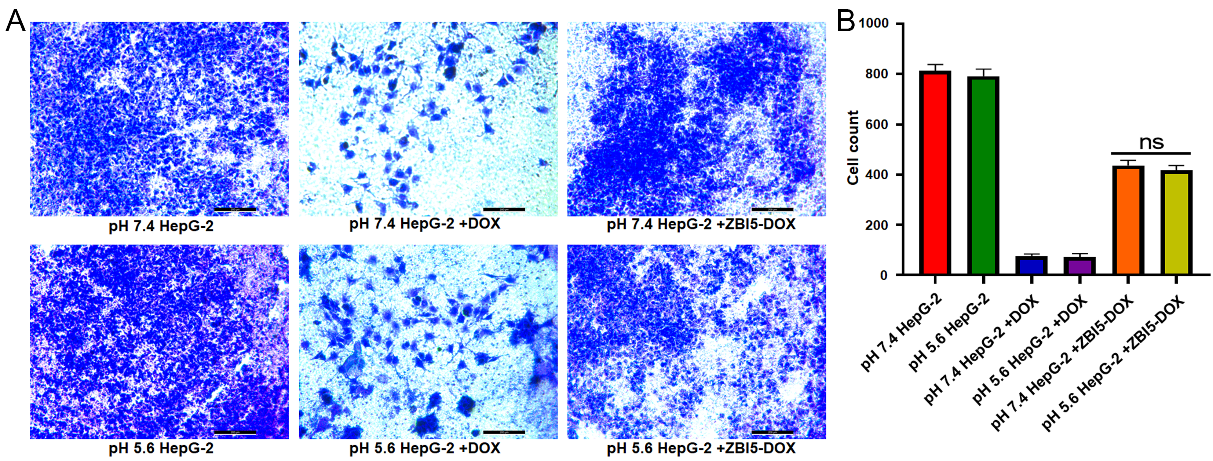


**FIGURE 27 |** Transwell invasion assays of control HepG-2 cells. (A) Images of HepG-2 cells treated with free DOX and ZBI5-DOX in pH 5.6 and pH 7.4. Untreated cells were used as controls. (B) Corresponding quantitative analysis of the cell counts. The data was presented as means ± SD, and ns indicates no significance.
